# Molecular Characterization of Novel Mycoviruses in Seven *Umbelopsis* Strains

**DOI:** 10.3390/v14112343

**Published:** 2022-10-25

**Authors:** Tünde Kartali, Nóra Zsindely, Ildikó Nyilasi, Orsolya Németh, Gergő Norbert Sávai, Sándor Kocsubé, Zoltán Lipinszki, Roland Patai, Krisztina Spisák, Gábor Nagy, László Bodai, Csaba Vágvölgyi, Tamás Papp

**Affiliations:** 1 ELKH-SZTE Fungal Pathogenicity Mechanisms Research Group, University of Szeged, 6726 Szeged, Hungary; 2 Department of Biochemistry and Molecular Biology, Faculty of Science and Informatics, University of Szeged, 6726 Szeged, Hungary; 3 Department of Microbiology, Faculty of Science and Informatics, University of Szeged, 6726 Szeged, Hungary; 4 MTA SZBK Lendület Laboratory of Cell Cycle Regulation, Institute of Biochemistry, Biological Research Centre, Eötvös Loránd Research Network (ELKH), 6726 Szeged, Hungary; 5 Neuronal Plasticity Research Group, Institute of Biophysics, Biological Research Centre, 6726 Szeged, Hungary; 6 Theoretical Medicine Doctoral School, University of Szeged, 6722 Szeged, Hungary

**Keywords:** dsRNA, mycovirus, *Totiviridae*, virus particles, *Umbelopsis*, Mucoromycota

## Abstract

The presence of viruses is less explored in Mucoromycota as compared to other fungal groups such as Ascomycota and Basidiomycota. Recently, more and more mycoviruses are identified from the early-diverging lineages of fungi. We have determined the genome of 11 novel dsRNA viruses in seven different *Umbelopsis* strains with next-generation sequencing (NGS). The identified viruses were named Umbelopsis ramanniana virus 5 (UrV5), 6a (UrV6a); 6b (UrV6b); 7 (UrV7); 8a (UrV8a); 8b (UrV8b); Umbelopsis gibberispora virus 1 (UgV1); 2 (UgV2) and Umbelopsis dimorpha virus 1a (UdV1a), 1b (UdV1b) and 2 (UdV2). All the newly identified viruses contain two open reading frames (ORFs), which putatively encode the coat protein (CP) and the RNA-dependent RNA polymerase (RdRp), respectively. Based on the phylogeny inferred from the RdRp sequences, eight viruses (UrV7, UrV8a, UrV8b, UgV1, UgV2, UdV1a, UdV1b and UdV2) belong to the genus *Totivirus*, while UrV5, UrV6a and UrV6b are placed into a yet unclassified but well-defined *Totiviridae*-related group. In UrV5, UgV1, UgV2, UrV8b, UdV1a, UdV2 and UdV1b, ORF2 is predicted to be translated as a fusion protein via a rare +1 (or −2) ribosomal frameshift, which is not characteristic to most members of the *Totivirus* genus. Virus particles 31 to 32 nm in diameter could be detected in the examined fungal strains by transmission electron microscopy. Through the identification and characterization of new viruses of Mucoromycota fungi, we can gain insight into the diversity of mycoviruses, as well as into their phylogeny and genome organization.

## 1. Introduction

Viruses have been identified in all main groups of fungi [1,2]. However, the vast majority of them were detected in members of the Ascomycota and Basidiomycota phyla, while the mycovirus harboring of basal fungi, such as those in the phylum Mucoromycota, have remained less explored [3]. Some recent studies suggest that the rate of virus carriage can also be high in these groups [3,4,5]. Most viruses described so far in the Mucoromycota have a linear double-stranded RNA genome and belong to the *Totiviridae* family [4,5]. Within this family, five genera, i.e., *Giardiavirus*, *Leishmaniavirus*, *Trichomonasvirus*, *Totivirus* and *Victorivirus* have been discerned, among which *Totivirus* and *Victorivirus* contain the majority of mycoviruses. Recently, two other genera have been proposed, i.e., *Artivirus*, which contain viruses from arthropods and fishes, and *Insevirus*, which contain viruses from insects [6]. Furthermore, phylogenetic studies have revealed several virus genomes, including mycoviruses, which cannot be associated with the currently accepted genera and are considered as yet unclassified totiviruses [4,5]. Viruses in the *Totiviridae* family have bicistronic, nonsegmented dsRNA genomes of 4.6–7.0 kilobases (kb), which are packaged into icosahedral virus particles with an average diameter of 40 nm [1]. Their genome most frequently contains two larger, mostly overlapping, open reading frames (ORFs), among which are the 5′ proximal ORF codes for the capsid protein (CP), while the 3’ proximal ORF corresponds to the RNA-dependent RNA polymerase (RdRp) [1].

The genus *Umbelopsis* belongs to the order Umbelopsidales, a sister group of Mucorales within the basal fungal phylum Mucoromycota [7]. The genus presently contains 16 species [8]. Some of them are root endophytes, while others are saprotrophic soil-dwelling organisms [7,9,10]. They are oleaginous fungi and have practical importance as producers of oils and fatty acids [11,12].

Vágvölgyi et al. (1993, 1998) were the first to investigate the virus harboring of the genus *Mucor*, during which four dsRNA fragments were detected in an isolate of *Mucor ramannianus*, which is now considered *U. ramanniana* [13,14]. Later, four dsRNA viruses, i.e., Umbelopsis ramanniana virus 1–4 (UrV1–4) belonging to the *Totiviridae* family were described in the same isolate [4]. In the same study, 24 Umbelopsis isolates representing 11 species were also screened, and the presence of multiple dsRNA elements was reported in five *Umbelopsis* species, suggesting that virus harboring might be common in this fungal group [4].

In the present study, six further isolates of *U. ramanniana* were examined for dsRNA carrying. Among them, two contained dsRNA elements. Thus, altogether, seven virus-harboring fungal strains were included in a detailed examination, during which the genome sequence of the identified mycoviruses were determined using whole genome sequencing (WGS), and the presence of virus particles were proven.

## 2. Materials and Methods

### 2.1. Fungal Strains and Cultivation

The 30 *Umbelopsis* strains involved in the study are presented in Supplementary Table S1. Strains were maintained on malt extract agar slants (MEA; 0.5% malt extract, 0.5% yeast extract, 1% glucose, and 2% agar) at 4 °C. Mycelia for dsRNA and virus particle isolation were grown in yeast extract–glucose broth (YEG; 1% glucose, 0.5% yeast extract) at 25 °C for 4 days on an orbital shaker at 150 rpm.

### 2.2. Purification of Nucleic Acids

To screen the strains for the presence of dsRNA elements, the total nucleic acid extraction method of Leach et al. [15] was used with modifications [5]. To purify the dsRNA from the total nucleic acid samples produced with the abovementioned method of Leach et al. [15] for NGS and “full-length amplification of cDNA” (FLAC), a previously described CF-11 cellulose chromatography method [5] was used. dsRNA molecules were separated by electrophoresis on 0.8% agarose/TAE (40 mMTris/acetic acid, 1 mM EDTA, pH 7.6) horizontal gels and visualized in UV after ethidium bromide (0.5 µg/mL) staining. Separated molecules were recovered from the gel using the Zymoclean Gel RNA Recovery Kit (Zymo Research, Irvin, CA, USA) according to the manufacturer’s instructions. The nature of the detected dsRNA elements was confirmed by their resistance to DNase I (Thermo Scientific, Waltham, MA, USA) and S1 nuclease (Thermo Scientific, Waltham, MA, USA) digestions carried out according to the recommendations of the manufacturers.

### 2.3. cDNA Synthesis and Sequencing of the dsRNA Molecules

For synthesis and amplification of cDNAs from dsRNA fragments, the FLAC method was used as described by Maan et al. 2007 [16] and modified by Darissa et al. 2010 [17]. Briefly, the PC3-T7 loop primer [18] was ligated to the purified dsRNA molecules; then, the primer-ligated dsRNA fragments were purified with the RNA Clean and Concentrator-5 kit (Zymo Research, Irvin, CA, USA), according to the manufacturer’s protocol. Denaturation and cDNA synthesis were performed as described previously [17]. Amplification of the cDNA was performed using a 1.25 µM PC2 primer [18] and 1 unit of the Phusion High-Fidelity DNA Polymerase (Thermo Scientific, Waltham, MA, USA), as described previously [4]. In some cases, the amplification of the cDNA was performed using a 0.5 µM PC2 primer [18] and 0.5 µM specific primer designated for the adequate sequence segment (Supplementary Table S2). Amplified DNA fragments were purified after agarose gel electrophoresis using the Zymoclean Large Fragment DNA Recovery Kit (Zymo Research, Irvin, CA, USA). Purified products were then cloned into the pJET1.2/Blunt vector (CloneJET PCR Cloning Kit, Thermo Scientific, Waltham, MA, USA). Sequences of the inserts were determined by the Eurofins Genomics Germany GmbH.

### 2.4. Generation of RNA Sequencing Library, NGS Sequencing and Bioinformatic Analysis

Indexed sequencing libraries were prepared from 80 ng viral genomic RNA samples using the NEBNext Ultra II Directional RNA Library Prep Kit for Illumina (New England Biolabs, San Diego, CA, USA) with NEBNext Multiplex Oligos for Illumina following the manufacturer’s protocol for use with purified mRNA or rRNA-depleted RNA. The main steps of this protocol, in short, are: fragmentation of RNA followed by synthesis of double-stranded cDNA, repair of cDNA ends, the addition of indexed adaptors to cDNA ends by ligation and limited PCR, and purification of the amplified libraries with NEBNext Sample Purification Beads. In the case of *U. gibberispora* CBS 109328, *U. angularis* CBS 603.68, *U. dimorpha* CBS 110039 and *U. versiformis* CBS 473.74 samples, the libraries were amplified for 10 PCR cycles, and fragment sizes of 200–300 bp were selected. In the case of *U. ramanniana* CBS 478.63 and *U. ramanniana* CBS 243.58 samples, 12 PCR cycles were applied and fragment sizes of 200–500 bp were selected. Sequencing library concentrations and fragment size distributions were determined by capillary gel electrophoresis with a 2100 Bioanalyzer (Agilent, Santa Clara, CA, USA) instrument using an Agilent DNA 1000 Kit. Libraries of shorter (*U. gibberispora* CBS 109328, *U. angularis* CBS 603.68, *U. dimorpha* CBS 110039 and *U. versiformis* CBS 473.74) or longer (*U. ramanniana* CBS 478.63 and *U. ramanniana* CBS 243.58) fragment sizes were pooled separately. After denaturing pooled libraries were sequenced in an Illumina MiSeq instrument (Illumina, Inc., San Diego, CA, USA), the pool of shorter (200–300 bp) libraries were sequenced using the MiSeq Reagent Kit v3-150, the pool of longer (200–500 bp) libraries were sequenced using the MiSeq Reagent Nano Kit v2-500, generating 2 × 75 bp or 2 × 250 bp paired-end sequence reads, respectively.

FASTQ sequence files were generated by GenerateFASTQ 1.1.0.64 application of Illumina BaseSpace. Adapter trimming and quality-dependent sequence trimming were done using TrimGalore with parameters: —paired —length 36 —q 20. To filter out host sequences, reads were aligned to subject sequence databases generated from corresponding *Umbelopsis* sp.-specific sequences available from the National Center for Biotechnology Information (NCBI) using the BLASTn algorithm.

### 2.5. Sequence and Phylogenetic Analysis

The sequences obtained were compared with those in NCBI nucleic acid and protein databases using the NCBI BLAST tool (http://blast.ncbi.nlm.nih.gov/Blast.cgi (accessed on 12 September 2022)). Putative amino acid sequences of the viral proteins were analyzed using the tools of the Expasy Bioinformatics Resource Portal (https://www.expasy.org/ (accessed on 12 September 2022)). Molecular weights were predicted using the Protein Molecular Weight program (https://www.bioinformatics.org/sms/prot_mw.html (accessed on 12 September 2022)). Possible RNA H-type pseudoknots were predicted using the DotKnot (https://dotknot.csse.uwa.edu.au/ (accessed on 12 September 2022)) [19] and visualized by the PseudoViewer web service (http://pseudoviewer.inha.ac.kr/ (accessed on 12 September 2022)) [20]. To determine the similarities of amino acid sequences, the EMBOSS Needle pairwise alignment tool at the website of the European Bioinformatics Institute (EMBL-EBI; https://www.ebi.ac.uk/Tools/msa/clustalo/ (accessed on 12 September 2022)) was used.

For the phylogenetic analysis, representative RdRp sequences of *Totiviridae*, *Chrysoviridae* and *Partitiviridae* were obtained from viruSite (http://www.virusite.org/index.php (accessed on 12 September 2022)). The dataset was supplemented by homologous hits of *U. ramanniana* RdRp sequences [4]. Sequences were aligned using MAFFT v. 7.453 [21] with the E-INS-i option. Best-fitting model for the maximum likelihood inference was selected by the ModelFinder [22] software of the IQ-TREE v. 1.6.12 package [23] based on the Bayesian Information Criterion [24]. The maximum likelihood analysis was conducted with IQ-TREE v. 1.6.12 applying the VT+F+R6 model. Statistical support of the best tree was calculated with ultrafast bootstrap approximation [25] in 5000 replicates.

The *Umbelopsis* phylogeny was inferred using sequences of the ITS region and part of the Mini-chromosome maintenance complex component 7 (MCM7). Alignments corresponding to the two regions were concatenated and partitioned. The dataset consisted of three partitions (rDNA, ITS1-ITS2 and MCM7). Model testing was performed on the partitioned dataset using the built-in model selection software of IQ-TREE v. 1.6.12. Best-fit models for the partitions were TPM2+F+G4 for the rDNA, TIM2e+G4 for the ITS region and TPM2u+F+G4 for the MCM7 region. Statistical support of the phylogenetic tree that best fits the dataset was tested with ultrafast bootstrap sampling with 5000 replicates.

### 2.6. Hybridization Analysis

dsRNA molecules and control plasmids were separated by electrophoresis on 1.0% agarose/TAE (40 mM Tris/acetic acid and 1 mM EDTA, pH 7.6) horizontal gels. Separated dsRNAs were denatured by rinsing the gel slides in 0.05 M NaOH and 0.15 M NaCl buffer for 30 min and neutralized in 1 M Tris-HCl and 1.5 M NaCl buffer (pH 7.5) for 2 × 20 min, as described by Hong et al. (1998) [26]. DNA samples were denatured in 0.5 M NaOH and 1.5 M NaCl buffer and neutralized in 0.5 M Tris and 1.5 M NaCl buffer (pH 7.5) [27]. Gel slides were blotted onto a positively charged nylon membrane (Amersham Hybond-N+, GE Healthcare) using 2× SSC buffer. After allowed to dry at room temperature, samples were immobilized with UV-crosslinking. Blots were hybridized with DIG-labeled CP and RdRp oligonucleotide probes in hybridization buffer (0.9 M NaCl, 1% SDS, 10% dextran sulfate) containing 5 µg/mL salmon sperm DNA (Invitrogen) (Supplementary Table S2). Probes were obtained by PCR from the corresponding DNA templates in the presence of digoxigenin-UTP (DIG DNA Labeling Mix, Roche, Sigma-Aldrich, Dorset, UK) using the DreamTaq polymerase (Thermo Scientific, Waltham, MA, USA). Primers applied to amplify the probes are listed in Supplementary Table S2. Hybridization was followed by immunological detection using alkaline phosphatase-conjugated anti-digoxigenin antibody (Roche, Sigma-Aldrich, Dorset, UK), according to the manufacturer’s instructions.

### 2.7. Examination of Virus Particles

Virus particles were isolated from 30 g frozen mycelium using the method of Lot et al. (1972) [28] followed by a discontinuous sucrose density gradient (10 to 40% (*w*/*v*) sucrose in PBS) centrifugation as described earlier [5]. The purified samples were negatively stained as described earlier [4] and examined with a JEM-1400 Flash transmission electron microscope (JEOL, Tokyo, Japan). Samples were systematically screened at 30,000× magnification to detect the virus particles on the grid. Then, particles were recorded at 50,000–120,000× magnification with a 16 MP Matataki Flash scientific complementary metal–oxide semiconductor (sCMOS) camera (JEOL). Quantitative analysis to determine the size of the particles was performed using the built-in measurement tools of the electron microscope, then data expressed as mean ± standard error of the mean.

## 3. Results

### 3.1. dsRNA Harboring in the Genus Umbelopsis

**Figure 1:**
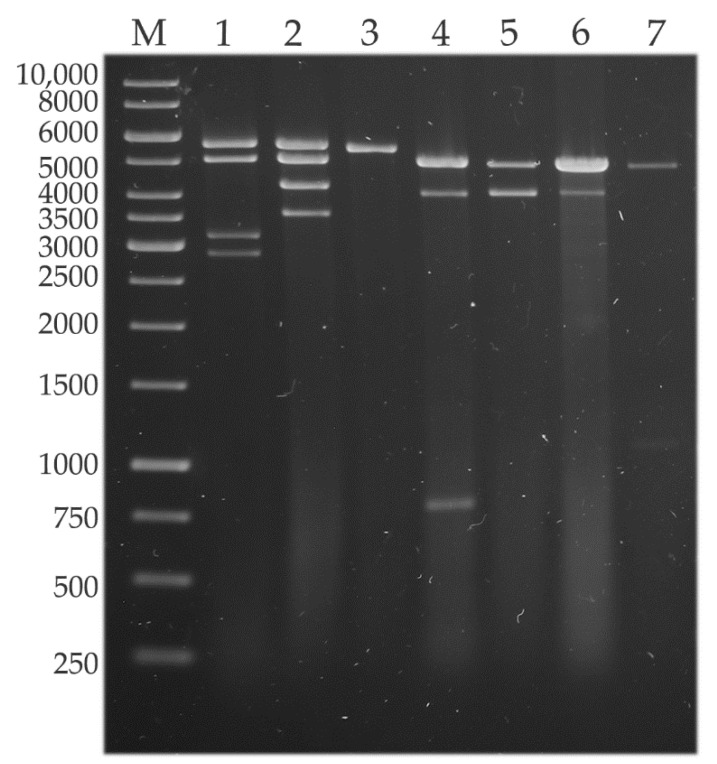
Gel electrophoresis pattern of the detected dsRNA genomes in seven mycovirus-harboring *Umbelopsis* strains. Lane M, GeneRuler 1 kb DNA Ladder (Thermo Scientific); Lane 1, *Umbelopsis ramanniana* NRRL 1296; Lane 2, *Umbelopsis ramanniana* CBS 478.63; Lane 3, *Umbelopsis ramanniana* CBS 243.58; Lane 4, *Umbelopsis gibberispora* CBS 109328; Lane 5, *Umbelopsis angularis* CBS 603.68; Lane 6, *Umbelopsis dimorpha* CBS 110039; Lane 7, *Umbelopsis versiformis* CBS 473.74.

The presence of dsRNA elements was confirmed in five strains previously [4]. In the present study, they were found in a further two *U. ramanniana* strains (Figure 1). The seven dsRNA harboring strains corresponded to the 23% of the tested *Umbelopsis* isolates (see Supplementary Table S1). For all previously tested strains, we achieved the same dsRNA patterns in this study. An approx. 5.0-kb fragment could be observed in almost all isolates. Out of the six tested *U. ramanniana* isolates, three proved to be dsRNA-harboring. The pattern observed in the *U. ramanniana* strain CBS 478.63 was similar to that described previously for NRRL 1296 containing the same sized 5.3- and 5.0-kb fragments (Figure 1), while CBS 243.58 contained only a single 5.3-kb fragment.

### 3.2. Whole Virus Genomes Identified in the Umbelopsis Isolates

Viral genome sequences were determined by NGS and assembled using SPAdes application with the –careful parameter. The sequencing depth (mean, median, minimal and maximal values) of the viral genome assemblies was the following: CBS 109328: 4629 bp contig 216/215/1/471, 4622 bp contig 48.38/46/1/158; CBS 603.68: 4613 bp contig 31/16/1/318, 3846 bp contig 408.2/48/1/7219; CBS 110039: 4753 bp contig 3894/3345/1/11,020, 4629 bp contig 344.6/287/1/1038; CBS 473.74: 221.1/48/1/3224; CBS 478.63: 4977 bp contig 117.2/111/1/224, 4619 bp contig 32.58/34/1/63, 4614 bp contig 18.66/19/1/38 and CBS 243.58: 31.12/32/1/66. The identified sequences were deposited to the NCBI database (accession no.: OM931137, OM931139, OM931138, OM931134, OM931135, OM931140, OM931130, OM931133, OM931131, OM931132 and OM931136).

NGS revealed 11 novel virus genomes in the seven dsRNA containing *Umbelopsis* strains (Table 1).

**Figure 2:**
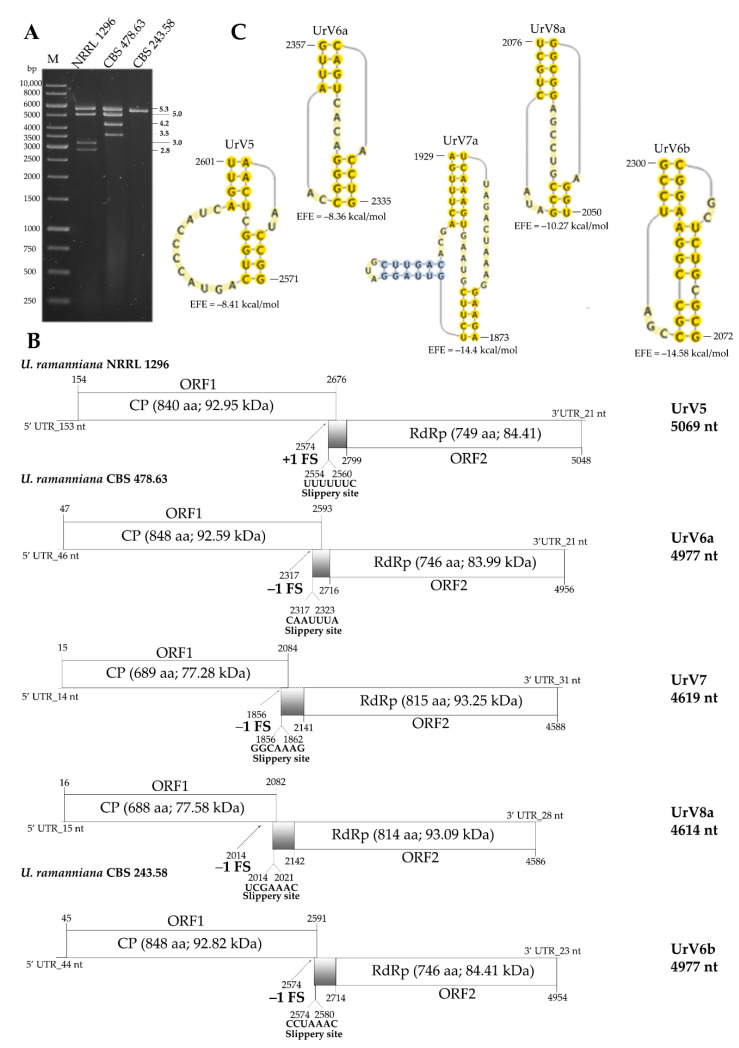
Gel electrophoresis pattern and genomic organization of the detected dsRNA genomes in three mycovirus-harboring *Umbelopsis ramanniana* strains. (**A**) Agarose gel electrophoresis of dsRNA fragments purified from the mycovirus-harboring *U. ramanniana* strains. Lane M, GeneRuler 1 kb DNA Ladder (Thermo Scientific); Lane 1, *Umbelopsis ramanniana* NRRL 1296; Lane 2, *Umbelopsis ramanniana* CBS 478.63; Lane 3, *Umbelopsis ramanniana* CBS 243.58. The sizes (kbp) of the detected dsRNA molecules and the corresponding virus genomes are indicated as well. (**B**) Genomic organization of the detected dsRNA genomes in *Umbelopsis ramanniana* NRRL 1296, *Umbelopsis ramanniana* CBS 478.63 and *Umbelopsis ramanniana* CBS 243.58, respectively, showing the putative open reading frames (ORFs). Gray boxes indicate the possible beginning of the fusion protein by the corresponding frameshifting and the spacer region. Position and sequence of the potential slippery sites is also shown. Abbreviations: CP, coat protein; RdRp, RNA-dependent RNA polymerase. UrV5, Umbelopsis ramanniana virus 5; UrV6a, Umbelopsis ramanniana virus 6a; UrV7, Umbelopsis ramanniana virus 7; UrV8a, Umbelopsis ramanniana virus 8a; UrV6b, Umbelopsis ramanniana virus 6b. (**C**) The pseudoknot structure downstream of the putative frameshift site of UrV5, UrV6a, UrV7, UrV8a and UrV6b. The RNA H-type pseudoknots were predicted by the DotKnot program [19] and drawn by the PseudoViewer program [20]. EFE (kcal/mol) indicates the estimated free energy.

**Figure 3:**
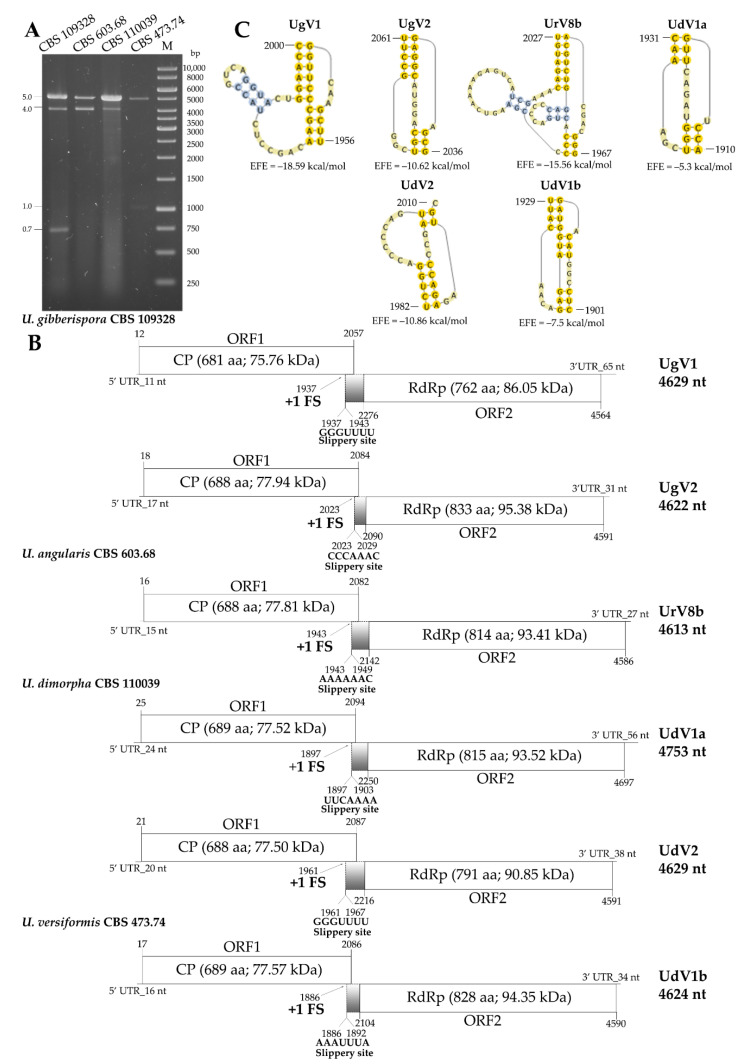
Gel electrophoresis pattern and genomic organization of the detected dsRNA genomes in three mycovirus-harboring *Umbelopsis* strains. (**A**) Agarose gel electrophoresis of dsRNA fragments purified from the mycovirus-harboring *Umbelopsis* strains. Lane 1, *Umbelopsis gibberispora* CBS 109328; Lane 2, *Umbelopsis angularis* CBS 603.68; Lane 3, *Umbelopsis dimorpha* CBS 110039; Lane 4, *Umbelopsis versiformis* CBS 473.74; Lane M, 1 kb DNS-marker Lane M, GeneRuler 1 kb DNA Ladder (Thermo Scientific). The sizes (kbp) of the detected dsRNA molecules and the corresponding virus genomes are indicated as well. (**B**) Genomic organization of the detected dsRNA genomes in *Umbelopsis gibberispora* CBS 109328, *Umbelopsis angularis* CBS 603.68, *Umbelopsis dimorpha* CBS 110039 and *Umbelopsis versiformis* CBS 473.74, respectively, showing putative open reading frames (ORFs). The gray boxes indicate the possible beginning of the fusion protein by the corresponding frameshifting and the spacer region. Position and sequence of the potential slippery site is also shown. Abbreviations: CP, coat protein; RdRp, RNA-dependent RNA polymerase; UgV1, Umbelopsis gibberispora virus 1; UgV2, Umbelopsis gibberispora virus 2; UrV8b, Umbelopsis ramanniana virus 8b; UdV1a, Umbelopsis dimorpha virus 1a; UdV2, Umbelopsis dimorpha virus 2; UdV1b, Umbelopsis dimorpha virus 1b. (**C**) The pseudoknot structure downstream of the putative frameshift site of UgV1, UgV2, UrV8b, UdV1a, UdV2 and UdV1b. The RNA H-type pseudoknots were predicted by the DotKnot program [19] and drawn by the PseudoViewer program [20]. EFE (kcal/mol) indicates the estimated free energy.

Predicted genome organization of the new viruses, including the position and length of the UTR sequences and the encoded ORFs, as well as the size and predicted molecular weight of the proteins encoded by the genomes are presented in Figure 2 and Figure 3 (predicted secondary structures of the 5′ and 3′ UTRs are also presented in Supplementary Figure S1). All genomes are undivided and contain two overlapping open reading frames, ORF1 and ORF2 encoding a CP and a RdRp, respectively. Relatively short UTR sequences were detected in the genomes as their length varied between 11 and 153 and 21 and 65 nt for the 5′ and 3′ UTRs, respectively. In each case, ORF1 and ORF2 were predicted to be translated as a fusion protein via a ribosomal frameshift. For each genome, a possible slippery heptamer and an H-type pseudoknot facilitating the programmed ribosomal frameshifting could be proposed in the overlapping region, as presented in Figure 2 and Figure 3. In each case of the newly identified mycoviruses, BLASTp homology search with the corresponding fragment sequence in the NCBI GenBank revealed a highest degree of identity with the CP and RdRp of viruses in the *Totiviridae* family (Supplementary Table S3).

The virus content of the *U. ramanniana* NRRL 1296 isolate was previously investigated in detail, and full genomes of four viruses named UrV1–4 were determined by the FLAC method [4]. In the present study, NGS sequencing was used to reexamine the virus genome sequences in this strain. The four previously described genomes of UrV1–4 could be detected by this method too. Additionally, NGS sequencing revealed a fifth, 5069-nt-long genome. The corresponding novel virus was named Umbelopsis ramanniana virus 5 (UrV5) (Table 1). Interestingly, ORF1 and ORF2 were predicted to be translated as a fusion protein via a rare +1 (or −2) ribosomal frameshift (Figure 2B).

In *U. ramanniana* CBS 478.63, three dsRNA genomes could be determined with 4977-, 4619- and 4614-nt in length, and the corresponding three viruses were named Umbelopsis ramanniana virus 6a (UrV6a), Umbelopsis ramanniana virus 7 (UrV7) and Umbelopsis ramanniana virus 8a (UrV8a), respectively (Table 1, Figure 2B).

In the case of *U. ramanniana* CBS 243.58, a single, 4977-nt-long virus genome was found (Figure 2B). The identified genome showed 83.2% identity with the UrV6a genome at the nucleotide level, while the CP and RdRp proteins of the two viruses proved to be identical by 95.8 and 95%, respectively. Accordingly, this novel virus found in the strain CBS 243.58 was named to Umbelopsis ramanniana virus 6b (UrV6b), indicating that UrV6a and UrV6b are closely related and can be regarded as two versions of the same virus.

Two virus genomes with 4629 and 4622 nt in length were detected in *U. gibberispora* CBS 109328, and the viruses were named as Umbelopsis gibberispora virus 1 (UgV1) and Umbelopsis gibberispora virus 2 (UgV2), respectively. For both viruses, ORF1 and ORF2 were predicted to be translated as a fusion protein via a +1 (or −2) ribosomal frameshift (Figure 3B).

In *U. angularis* CBS 603.68, a single 4613-nt genome was determined (Figure 3B). The identified genome is most likely a variant of the UrV8 genome, because its sequence shows 79.6% identity with that of the UrV8a at the nucleic acid level. Moreover, amino acid sequences of the CP and RdRp of the two viruses proved to be identical by 95.5% and 92.4%, respectively. Therefore, the virus identified in *U. angularis* CBS 603.68 was described as Umbelopsis ramanniana virus 8b (UrV8b).

In the sample of *U. dimorpha* CBS 110039, two mycovirus genomes were revealed, and the corresponding viruses were named as Umbelopsis dimorpha virus 1a (UdV1a) and Umbelopsis dimorpha virus 2 (UdV2) (Table 1, Figure 3B).

In the *U. versiformis* CBS 473.74 strain, NGS identified a 4624-nt viral genome, which showed 91.4% (CP) and 80.6% (RdRp) identity with the corresponding UdV1a sequences at the amino acid level. Based on this, we named it Umbelopsis dimorpha virus 1b (UdV1b) and regarded as a variant of the UdV1 virus (Figure 3B).

### 3.3. Phylogenetic Analysis

**Figure 4:**
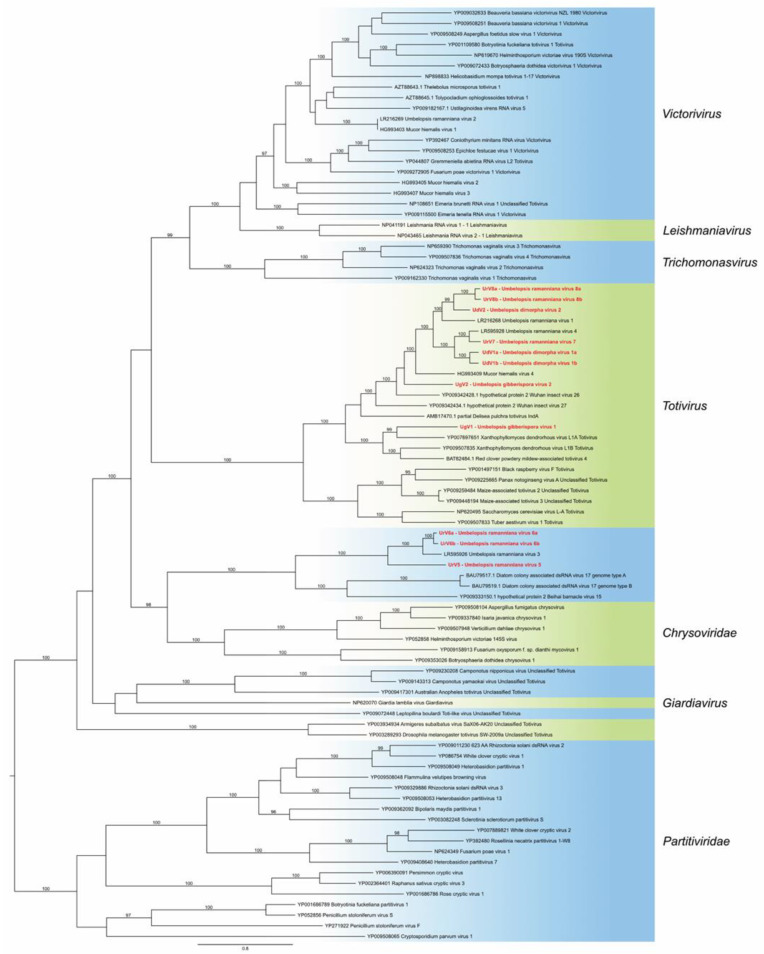
Phylogeny based on the RdRp amino acid sequences from representative members of the families *Totiviridae*, *Chrysoviridae* and *Partitiviridae*. The accession numbers are indicated on the tree. The resulting tree indicates that UdV1a, UdV1b, UdV2, UrV7, UrV8a, UrV8b, UgV1 and UgV2 are members of the genus *Totivirus*, while UrV5, UrV6a and UrV6b are seated in the well-defined but yet unclassified clade, which is closely related to the *Chrysoviridae* clade. Only bootstrap values higher than 95% are shown. The novel *Umbelopsis* mycoviruses are indicated with red letters.

Based on the RdRp amino acid sequences of the newly identified genomes and representative members from the viral families *Totiviridae*, *Chrysoviridae* and *Partitiviridae*, a phylogeny was inferred using the ML method (Figure 4). This analysis demonstrated that all 11 novel viruses belong to the *Totiviridae* family. Eight viruses, i.e., UdV1a, UdV1b, UdV2, UrV7, UrV8a, UrV8b, UgV1 and UgV2, are part of the clade representing the genus *Totivirus*. Among them, UdV1a, UdV1b, UdV2, UrV7, UrV8a, UrV8b and UgV2 proved to be closely related to the previously described UrV1, UrV4 [4] and Mucor hiemalis virus 4 [5]. UgV1 seated in another subclade of the *Totivirus* clade together with other mycoviruses—among others, Xanthophyllomyces dendrorhous virus L 1A. UrV5, UrV6a and UrV6b are in the same clade as the earlier described UrV3 [4]. These viruses form a well-defined but yet unclassified clade together with viruses of nonfungal organisms, such as Beihai barnacle virus 15 and Diatom colony associated dsRNA virus 17 A and B.

### 3.4. Hybridization Analysis of the dsRNA Patterns

A hybridization analysis revealed that the UrV5 genome corresponds to the 5.3-kb fragment of the dsRNA pattern of the *U. ramanniana* NRRL 1296 isolate (Supplementary Figure S2).

**Figure 5:**
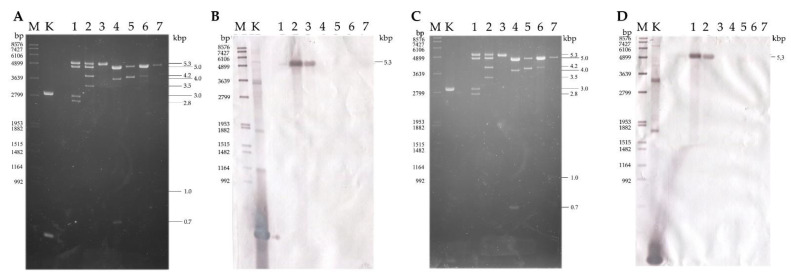
Northern blot analysis of dsRNA molecules purified from the mycovirus-harboring *Umbelopsis ramanniana* CBS 478.63 and CBS 243.58 strains using the UrV6a CP and RdRp probes, which were amplified with primers presented in Supplementary Table S2. Panels (**A**,**C**): agarose gel electrophoresis of the dsRNA molecules purified from the mycovirus-harboring *Umbelopsis* strains. Lane K, control plasmid containing the corresponding PCR amplicon of virus genomes; Lane M, DIG-labeled DNA Molecular Weight Marker VII (Roche Lane 1, *Umbelopsis ramanniana* NRRL 1296; Lane 2, *Umbelopsis ramanniana* CBS 478.63; Lane 3, *Umbelopsis ramanniana* CBS 243.58; Lane 4, *Umbelopsis gibberispora* CBS 109328; Lane 5, *Umbelopsis angularis* CBS 603.68; Lane 6, *Umbelopsis dimorpha* CBS 110039; Lane 7, *Umbelopsis versiformis* CBS 473.74. Right numbers indicate the sizes (kbp) of the detected dsRNA molecules. Panels (**B**,**D**): Northern blot analysis of the dsRNA molecules extracted from *U. ramanniana* CBS 478.63 and CBS 243.58 strains using the UrV6a/b CP and UrV6a/b RdRp probes, respectively. Both probes, the UrV6a/b CP (**B**) and the UrV6a/b RdRp (**D**) gave strong hybridization signals to the largest, 5.3-kbp band purified from the *U. ramanniana* CBS 478.63 and CBS 243.58 strains, as well as the *Bgl*II digested control plasmid, which contains the corresponding PCR amplicon of the UrV6a/b CP and UrV6a/b RdRp probe sequences.

*U. ramanniana* CBS 478.63 had an electrophoretic pattern consisting of four fragments (Figure 2). During the hybridization experiments, the probes designed for the CP and RdRp of the UrV6a virus hybridized to the 5.3 kb fragment (Figure 5). It is worth mentioning that the fragment in the *U. ramanniana* CBS 243.58 isolate also gave a signal of a similar strength with the UrV6a probes demonstrating the high level of sequence identity between UrV6a and UrV6b (Figure 5) The probes designed for UrV7 and UrV8a viruses hybridized to the second, 5.0-kb fragment (Supplementary Figure S3). In the dsRNA pattern of *U. ramanniana* CBS 478.63, two smaller fragments can also be observed, for which we could not recover the sequence information.

In the case of *U. gibberispora* CBS 109328, probes designed for the CP and RdRp genes of UgV1 and UgV2 hybridized to the larger, 5.0-kb fragment (Supplementary Figure S4). For this isolate, we detected a smaller dsRNA fragment of 4.0 kb in size, which, according to our present investigation, we do not have any sequence information. Similarly, a third fragment of approx. 0.7 kb was also observed in the electrophoretic pattern. Although we did not manage to identify this fragment with NGS, a 661-nt segment could be isolated and sequenced using the FLAC method (Supplementary Data S1). However, the resulting sequence did not match any other sequences in the NCBI GenBank database either at the nucleotide or the amino acid level.

In *U. angularis CBS 603.68*, two dsRNA fragments were found, but we could only identify one viral genome, which was 4613 nt in size. Probes designed for UrV8b CP and RdRp genes hybridized with the larger 5.0-kb fragment (Supplementary Figure S5). A second, approximately 4.0-kb sized dsRNA was also detected by gel electrophoresis in this isolate (Figure 1). From this 4.0-kb dsRNA fragment, cDNA was generated by the FLAC technique, and the resulted clone was sequenced. Thus, a 3847-nt sequence was determined (Supplementary Data S1). The fragment contains a single ORF (from 1195 to 3645 nt), which was predicted to encode a putative, 816-aa hypothetical protein. However, the nucleic acid and the predicted amino acid sequence of this hypothetical protein did not show any similarities to known mycoviral sequences and other sequences in the GenBank by Blast queries.

In the case of the sample from *U. dimorpha* CBS 110039, we found that probes designed for both viruses (i.e., UdV1a and UdV2) hybridized to the 5.0-kb fragment (Supplementary Figure S6) The probe designed for the RdRp of UdV1a also gave a signal with the 5.0 kb fragments of *U. ramanniana* NRRL 1296, *U. ramanniana* CBS 478.63, *U. angularis* CBS 603.68 and *U. versiformis* CBS 473.74 isolates. The smaller 4.0-kb fragment detected during gel electrophoresis could also not be determined.

Probes designed for the coding regions of UdV1b hybridized to the 5.0-kb fragment of the isolate *U. versiformis* CBS 473.74 (Supplementary Figure S7). dsRNA pattern of this isolate also contained an approx. 1-kb fragment, which could be identified with the FLAC method (Supplementary Data S1). The exact size of the fragment was 1069 nt and proved to be a partial RdRp coding sequence, which showed 99.7% of identity with a 1070-nt section of the UrV4 genome. UrV4 was identified in the *U. ramanniana* NRRL 1296 isolate earlier [4]. Presumably, this could be residual or cryptic viral nucleic acid in the host fungus.

### 3.5. Diversity of Mycoviruses in the Genus Umbelopsis

**Figure 6:**
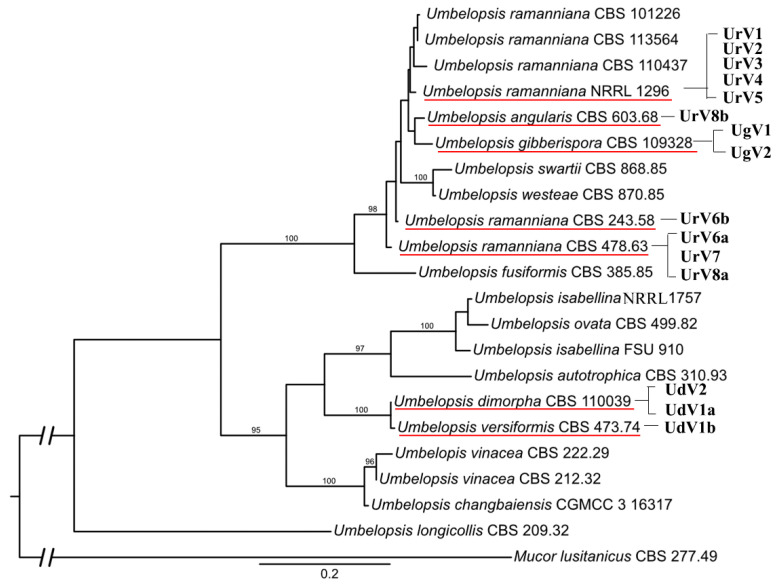
Comparison of the phylogeny of the tested *Umbelopsis* species inferred from the ITS and MCM7 sequences. Model testing was performed on the partitioned dataset using the built-in model selection software of IQ-TREE v. 1.6.12. Bootstrap values (%) indicated on branches were obtained with 5000 replicates. *Mucor lusitanicus* CBS 277.49 was used as the outgroup. Virus-carrying isolates have been marked with a red underline. UrV1, Umbelopsis ramanniana virus 1; UrV2, Umbelopsis ramanniana virus 2; UrV3, Umbelopsis ramanniana virus 3; UrV4, Umbelopsis ramanniana virus 4; UrV5, Umbelopsis ramanniana virus 5; UrV8b, Umbelopsis ramanniana virus 8b; UgV1, Umbelopsis gibberispora virus 1; UgV2, Umbelopsis gibberispora virus 2; UrV6b, Umbelopsis ramanniana virus 6b; UrV6a, Umbelopsis ramanniana virus 6a; UrV7, Umbelopsis ramanniana virus 7; UrV8a, Umbelopsis ramanniana virus 8a; UdV2, Umbelopsis dimorpha virus 2; UdV1a, Umbelopsis dimorpha virus 1a; UdV1b, Umbelopsis dimorpha virus 1b.

To get an idea about the viral diversity of the genus, the virus harboring feature was compared with the phylogeny of the examined *Umbelopsis* strains (Figure 6). In this phylogeny, the *Umbelopsis* genus is split to two large clades. Among them, the clade containing *U. ramanniana*, *U. angularis* and *U. gibberispora* has a much higher proportion of the virus-carrying strains than the clade containing *U. dimorpha* and *U. versiformis*. *U. dimorpha* CBS 110039 and *U. versiformis* CBS 473.74, which form a sister group on the tree carry variants of the same virus species (UdV1a and UdV1b). We also identified two genome variants of the same virus (UrV6a and UrV6b) in *U. ramanniana* CBS 478.63 and CBS 243.58 isolates, which are more closely related to each other than to the other *U. ramanniana* isolates. In other cases, however, we found that, despite the close phylogenetic relationship of the virus-carrying isolates, the identified virus genomes differed to a greater extent. This can be seen in the case of *U. angularis* CBS 603.68 and *U. gibberispora* CBS 109328 isolates. Moreover, the UrV8b virus was identified in the *U. angularis* CBS 603.68 strain, while UrV8a was detected in the more distantly related *U. ramanniana* CBS 478.63 isolate.

### 3.6. Detection of Virus Particles in the Virus-Harboring Umbelopsis Strains

**Figure 7:**
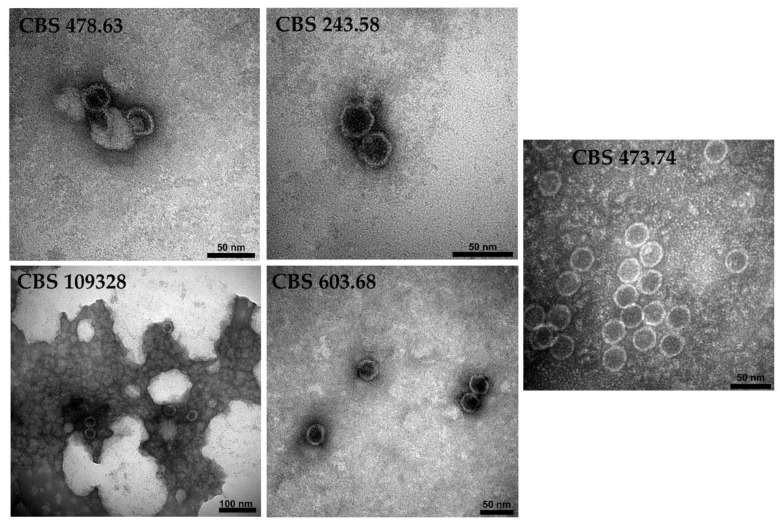
Virus-like particles detected in five mycovirus-harboring *Umbelopsis* strains. The virus particles were recovered by ultracentrifugation at 78,000× *g* for 12 h at 4 °C, which follows with sucrose gradient density centrifugation. Purified virus particles were negatively stained with 2% uranyl acetate in 50% ethanol for 5 min (3 times) and examined under a JEM-1400 Flash transmission electron microscope. VLPs about 31–32 nm in diameter were detected in the examined *Umbelopsis* strains.

The presence of virus-like particles (VLPs) in *U. ramanniana* NRRL 1296 was already reported [4]. In this study, transmission electron microscopy revealed the presence of isometric VLPs in the purified extracts of the other five mycovirus-harboring *Umbelopsis* strains (Figure 7). VLPs about 32 nm in diameter were detected in *U. ramanniana* CBS 478.63 and CBS 243.58, *U. gibberispora* CBS 109328 and *U. angularis* CBS 603.68, while the VLPs in *U. versiformis* CBS 473.74 proved to be about 31 nm in diameter. VLPs could not be detected in the *U. dimorpha* CBS 110039 strain.

## 4. Discussion

In our studies (i.e., Kartali et al. 2019 [4] and the present study), altogether, 30 *Umbelopsis* strains representing 11 species were screened for the presence of mycoviruses and 23% of the tested isolates proved to be virus-harboring. This proportion corresponds to the virus carriage frequencies found within Mucoromycota fungi by other studies. Myers et al. (2020) examined 71 isolates from 30 Mucoromycota genera, and the presence of viruses was detected in 19.7% of the tested isolates [3] Earlier, Papp et al. (2001) found a similar virus-harboring frequency (19%) for *Rhizopus* isolates [29].

All viruses identified in the present study proved to be the member of the *Totiviridae* family. Previously, four viruses were already described in *U. ramanniana* NRRL 1296, which also belonged to the *Totiviridae* [4]. Myers et al. (2020) also detected a Totivirus-like genome in a strain of *Umbelopsis nana* [3]. Among the newly described 11 viruses, eight belong to the genus *Totivirus* and three seated in a well-separated clade containing other non-classified Totivirus-like viruses (Figure 4). This unclassified group contains UrV3, which was earlier described from *U. ramanniana* [4] and viruses identified from nonfungal organisms, i.e., from invertebrates and diatoms [30,31]. Although this unclassified group contained viruses with undivided Totiviridae-type dsRNA genomes, it proved to be closely related to the *Chrysoviridae*, which is a family of viruses with segmented genomes typically consisting of four dsRNA segments [32]. The close phylogenetic relationship between *Totiviridae* and *Chrysoviridae* has been reported several times [4,32,33].

Within the genus *Totivirus*, seven newly identified viruses, i.e., UrV7, UrV8a, UrV8b, UgV2, UdV1a, UdV1b and UdV2, together with the earlier described UrV1 and UrV4 [4] form a monophyletic subclade containing viruses isolated exclusively from *Umbelopsis* strains. This subclade is closely related to MhV4 [5] and the Trichoderma koningiopsis totivirus 1 (TkTV1) [34].

UgV1 is located away from the former group and forms another subclade of *Totivirus* with Plasmopara viticola lesion associated toti 3 [35] and Xanthophyllomyces dendrorhous virus L1A [36]. Although we identified another virus, UgV2, from the same host (i.e., *U. gibberispora* CBS 109328), the similarity between the two genomes was minimal, which is supported by their location on the phylogeny (Figure 4).

When virus carriage was compared with the phylogeny of the fungal isolates (Figure 6), it was observed that isolates representing different species may carry the genome variants of the same virus, while closely related fungal isolates contain slightly related viruses. These observations raise the possibility of horizontal virus transmission among the different fungal isolates as has recently been increasingly assumed for other viruses and hosts too [34].

Among the seven tested strains, four isolates contain multiple viruses (Table 1). Moreover, presence of five virus genomes was proven in *U. ramanniana* NRRL 1296 [4]. The presence of multiple viruses in the same strain have been reported from several fungi, such as from *Aspergillus fumigatus* [37], or from the plant pathogenic fungus *Exobasidium* sp. [38]. In agreement with previous reports [3,4,5], our results suggest that mixed infections can commonly occur in Mucoromycota fungus.

Genome organization of the identified viruses show the typical features of the genomes in the genus *Totivirus*, i.e., an undivided dsRNA genome with two partially overlapping ORFs [1]. This arrangement suggests the translation of RdRp as a fusion protein [39]. Generally, it is proposed for the totiviruses that translation of the fusion protein is carried out via a programmed -1 ribosomal frameshift [39]. However, in seven novel genomes, i.e., in those of UrV5, UgV1, UgV2, UrV8b, UdV1a, Udv1b and UdV2, a +1(-2) ribosomal frameshift could be predicted. This type of frameshift is considered as a rare mechanism in *Totiviridae* and has been reported mainly in the genera *Trichomonas* and *Leishmaniavirus* [40,41]. It is worth mentioning that +1(−2) ribosomal frameshift was also proposed for the Mucor hiemalis virus 4 (MhV4) belonging to the genus *Totivirus* [5].

Approximately the same-sized VLPs, about 31 to 32 nm in diameter, could be observed in all examined *Umbelopsis* strains. Morphology and size of the detected VLPs are typical for the *Totiviridae* family [42]. In the case of *U. ramanniana* CBS 478.63 and *U. gibberispora* CBS 109328 with multiple mycoviruses, we detected one-sized VLPs, with 31 or 32 nm in diameter (Figure 7). This could be explained with the approximately same genome sizes of the corresponding viruses (Figure 2B and Figure 3B). In *U. dimorpha* CBS 110039, we did not detect any VLPs, which could be explained by the very low attendance of VLPs in this fungus.

In conclusion, we identified and described 11 novel *Totiviridae*-related viruses from five *Umbelopsis* species and could obtain an insight into the mycoviral diversity of this fungal genus. In four strains, the presence of multiple viruses was proven. Our results also suggest that a +1 ribosomal frameshift might not be so rare a process in the *Totiviridae* family as previously reported.

## Figures and Tables

**Table 1:** Novel viruses identified in *Umbelopsis* isolates.

Species	Collection Number	Substrate, Origin	Name of the Detected Virus; Abbreviation	NCBI Accession No. of the Genomes
*U. ramanniana*	NRRL 1296	Wisconsin, USA	Umbelopsis ramanniana virus 5; UrV5	OM931140
*U. ramanniana*	CBS 478.63	*Amanita* sp., Netherlands	Umbelopsis ramanniana virus 6a; UrV6a Umbelopsis ramanniana virus 7; UrV7 Umbelopsis ramanniana virus 8a; Ur8a	OM931130 OM931131 OM931132
*U. ramanniana*	CBS 243.58	Skin between toes, Netherlands	Umbelopsis ramanniana virus 6b; UrV6b	OM931133
*U. gibberispora*	CBS 109328	*Fagus crenata*, Japan	Umbelopsis gibberispora virus 1; UgV1 Umbelopsis gibberispora virus 2; UgV2	OM931134 OM931135
*U. angularis*	CBS 603.68	Soil, Netherlands	Umbelopsis ramanniana virus 8b; UrV8b	OM931136
*U. dimorpha*	CBS 110039	Soil, New Zealand	Umbelopsis dimorpha virus 1a; UdV1a Umbelopsis dimorpha virus 2; UdV2	OM931137 OM931138
*U. versiformis*	CBS 473.74	Soil, Victoria, Australia	Umbelopsis dimorpha virus 1b; UdV1b	OM931139

## Data Availability

All data generated or analyzed during this study are included in this article and its supplementary information files. The strains used are available from the Szeged Microbiological Collection (www.szmc.hu (accessed on 2 January 2021); email: pappt@bio.u-szeged.hu).

## Supplementary Materials

The following supporting information can be downloaded at: https://www.mdpi.com/article/10.3390/v14112343/s1: Table S1: The tested *Umbelopsis* strains. Table S2: List of primers applying for amplification of cDNAs and hybridization probes. Table S3: Amino acid sequence identities of the UrV5 CP, UrV5 RdRp, UrV6a CP, UrV6a RdRp, UrV7 CP, UrV7 RdRp, UrV8a CP, UrV8a RdRp, UrV6b CP, UrV6b RdRp, UgV1 CP, UgV1 RdRp, UgV2 CP, UgV2 RdRp, UgV8b CP, UgV8b RdRp, UdV1a CP, UdV1b RdRp, UdV2 CP, UdV2 RdRp, UdV1b CP and UdV1b RdRp. Data S1: Nonmycoviral nucleic acid sequences determined in the study. Figure S1: The predicted secondary structures of the 5′ and 3′ UTRs of the detected viruses. Figure S2–S7: Northern blot analysis of dsRNA molecules purified from the mycovirus-harboring Umbelopsis strains with probes designed using the UrV5, UrV7, UrV8a, UgV1, UgV2, UrV8b, UdV1a, UdV2 and UdV1b sequences, used in this work and taken from [5] and [8].
