# Supplementary material for: Molecular Characterization of Novel Mycoviruses in Seven Umbelopsis Strains
Source: Viruses. 2022 Oct 25;14(11):2343. doi: 10.3390/v14112343 (PMC9694724; doi:10.3390/v14112343)
Supplement: Supplementary file 1 [file viruses-14-02343-s001.zip › Supplementary Data 1_fasta.pdf]

>Umbelopsis *gibberispora* CBS 109328 – 631 nt
tcaaggtggctcagggacgtgccgcaggttcggcctcgcagaggaggattttggtgatgggcacaaagag
ggctgggctgagggccatccagtgtgagtgcctgacgctgagctgcggcgaggctaaggccaggctccagc
atgtcaactaccacaaactactacgcacggtaggacagccaggaacagcctgtctgaaacggttgagaagc
atcgactgtcaagggtgaagaatgagtgtgcatggtttcttgctgggtggcggccaagcaccagttgctag
aacactttggcaagcaggctcacattggttgaccacaaaggccggtaaagccgtggccactgagctagctc
tcagacatgtggcaccactggattggcaacgcgccatagggagttggggcaggccacgttctgcgcgagcg
gtgatagccaggtaggggctcctgcttgacctgcctgggtctgcagacatgtggtacgcaatggttggccg
tcgccgggcgatgggtccagcatgtatgccggacttgttgggaggtgaacaacaactaaaggttggcggtg
gaaatgccactgccattatgcaccgattggtgcacaacaaaacgaagggattcgtttcccccc

>Umbelopsis versiformis CBS 473.74 – 1069 nt
tatgcagttgagtgcggcagagtggacgaaacagtcaatatccgccaccatcgtaaacgacaatatggggacgaa
gacgcaatacaaaacaaacggcaccctaatgtctggctggcgtttaacaacctacatgaactcaattctcaatta
catatacacgaactattgaccaaggatacagaaagcacctaccaatcggtacacaacggtgatgatgtactgctc
ggggtcagaaacttcgatatagcgaggagggctgtatttaatgcggataagtacaatgtccgtttgcaacgcagc
aaatgcacgttcgggggtatagcggaatttctgcgtgttgaccgcgttcgaggtgatttcggtcagtatctgtca
aggaacgtagcaacgttaatgcatgccagaatagagtccaagctagccctaagcgtagtggacttggtcgaggca
tctgaagaacgtttgcgtgaattcatacagcgaggtggctcacctaagactgcggccaggttgaggagtatcgcg
tatgacagatattccaagatttatgaaacggacacagccactctctaccgtatcaaatattctcaccgtgtagca
gggggaatatctgatggcctcggagcacctattgatcaggtgataaataaggatcaagtcgggcggatagcggag
ctaccggactatctgccaggcatagcagactattcaaacgtgctcaagaagagtttaaatttgaatatggaagta
agtaaaatagcaaaacggatatacagcgccacactaaatgctgttaagttagaaaggactaaggttcacaccgag
gtcccagagaacatagaacaactgaaagtgtacagagctttgtataaagctcacagcgatgcaactgataatgct
gcatttggaaaggcgattctaacaggattcgtatttgacgtgctcagtcgtaatgacaaggcgaacacgttgatg
ggcatcttgtaccaatcgaaggacccgatgcaattgttaaaagtaatagcatgagtgggctgcgccacaaaaata
gcgcacctgtcacacgtca

>Umbelopsis angularis CBS 603.68 – 3847 nt
tgaaaatttaccgcaatctacctgctgtaaggtttttcaacttataatttcgtccaactctgtgtacaacttaat
tgataagcattcaaagcagtggtcgtcatacgtataaatccgtatgcctgctgttcccaataaacataactctac
aacaaagcatgatcaaatcaggtagcttaccaacagacaagtcaacatttgttgaccaccttgtgattagtgaaa
aagtgagaaggattattataagaccttatattgaggctcagacccatgtacatctgccatatgttaaaaaaccag
acatatggcagataattaaatctgccatattaactgagctgaatatcgacattcagaagttgtacgatgatgacg
ctgagtattcactaaaagtttgctacgaagtgttgctatcgagttgcggattaatgtcagaccgatgggattact
ataacattaaatctccattcgtattagggagtgcaaaaatgtctttcgatgtattgggaatacttgtagttactc
tatacttgcagagctgcgtgcaaagtggcgggaggattaatccgccgtcgggaagtagagccgacattgttcaag
gagtcagcaacgataaagaagtaacttgctttgtaagtataagtggcgacgtggtaatcaagttggagtcagcac
tagccgctatacacaggcgtagtgttggtaataacagttgtcttggtggacagttgaatggtatgtttattgcta
gtagacgtatgtctggtttagtagattctttggaggaaaggctccgaaccacaacgaacgaagtttggtatggtg
tccacactcagagtcaagctgaaaatactctgtacgatgcacttactgcattgggtttatctcattttaaaacgc
caggatcaaacgttatattgcctaagggcgggttggtactgaggacctctgtaaatgtaatagtcgtagtgtcag
aattagtgcagacgtcagcaagtgtttccgcaatgagatcatttgccgaggaaggcgataaacatgccaggacat
ctactatgttatttgttttgacgttatattgctcatcgtcttaggtccgccattagcagtaggactcgtccccga
agcttcagtaaaatcacatatagacccttcttctctgttcgcatgtatgttaggggttaggggataatcatgacg
atttttaaatccctttacgctaaagattggagttggtacgattttataaggttcagggtctacaatagtgagatc
acggttggctctaacaagttgtccattgacccgctggaagcagtggtcgaatcagtaagacatagagatttctac
gagcgaaagcttaatggcgttaatctgtgtccttcaggtattaattatagcggagacaaggccatacgtctagct
gtacccgtcttagattgggtcgcgattggttatatagcaatccaaattgaggacgcggacggtgtctcaattata
atgcaacacgtcggcggccggcccacagttggaaaaatttctggcacgatggctatgtgtgccactcagcgtgac
gatgtcacagtccacgttagtaagataccagctagctctgacaagcttacaggtgacgtaattacctcattaccg
ggtgtttacttaggttacgaccccagagatgaagtcaaagctaaaaaatttatacacgcgcgagttctaaaacct
cagctagggtttcacatggaagatggcgtccagggagatcatgatcgtgtgattgatcgtgtgtttactgcaaag
ctgttccaagatgaggtgcctctatcacctagctttaacgcgcaagagaggcctaataccatagaaagatcacct
tcacgcattagtgatagcccactcacaacgtgcaactccaagactagtgatagtgaaaccatgcctgagtcaagc
tacgtttggctcggttgtgacgcgtatggaagaacatcttacggcacttgtatcgacaacgaccatgaatgggta
aatgatgctgacttcattggaagaggcagcactgaaatcaatattgcaatagcagaactgcggtacagttgtcaa
gatactaccagtatctatgtatgtaaaacatgtacaacatataaaatgattagaagtagtctagacgctcagaga
caggaagatatgacatcttttcagaagtctacaccaaagcaagaagtgtgtacgagacacaacatccaggtgcat

ataaagcatcatgaccgtttccacagtctgtgtaatggtgttgcgatgtgcgactcgtgcggtataaagaaaaca
gggtgttatctgtgtggatcggaattgatccagcaagaaagagaattagcactcaagtatgctgagtcggataca
gactccgatgcgtatagcgtcatgagtgatggtatgatatgtgatcatagtgaggaccactgtacggatgaaaca
atacagcaagtttctgtcagaaaaaatactaacgagcctgaagatctagatttcggcgaatttaatagggaacac
gacactacctacccccagaccactaacgaagacctgacactatgcgagactctagagttgttgtatgatttaagt
gatgactttgctcaggcagcagttttacatgcaattgtgaaccctcggagtactaaaaatcatttcagctctgaa
gtggttgaggaggtattattagcagcgggcataagcttttcgatatgtagatatattaaagggaggactataagc
ttacgagacatcaacatcaatatccgcaaagctgttgaaatgaagctcgaacatatagattttgatggtgctatc
gacagatgtaagcgagaagcagagatgagcagtcaggcgatatcaagggaagaccaaaatttgataataagctta
tgttttaaggaagcctccccattaggaacacatatagttaagggtaaatctagtaatgatatgttggagtgtgaa
gtaaccccaaataagttgatcagcgagatggtctcaattctagctgttggcaacatcaagctgagtaaaaagaag
attgctagagctgggaagagggcggaagaacacgtaaatattagtaactgcggttgtatttatgctgatatcgca
atccagcgattactgggtgtgcttcagcctgagcacgtggggtatgatcagtatgaggtaaagacggtagtgaaa
tttaaggatctgataacgaaacttgcgcgtaggttttcagtagaggaaaaggtgatattatttacgatatgtact
actatatctagctacaggtgcaatgtggctctgtataagcagtgtgggaaatcgagaagttcagagcgtatggta
gcaaaaagtaggaagagagagaatattgtattcgataggtccgttgcgaatttcacacagaacggagtaagtaat
gctatgattgaaaaacctcataaagggttatgtaaagtcaagttttggaaaaaaatactgaaactagaaatcggt
aggtggctagggttgaatgcaagaggtaggaataagagactagccgggcaatgtgaaaaacagctaaaactcgca
aaggctatgatcacaacttctagatatatatcactggacatatagaaatggcggccaccccggctggaaataggg
gttagggcgcaatagacacgacacaaatgttcaaggttatgcattctctgggcgactcaatgcgagttcacagcc
cagacagtgtagtgaggaaagtcacacgggaagcatgctgttgtagccagtgtgcgagccggaaaggttaaaata
gagcaggatcccgggaattcgg
