# Supplementary material for: Molecular Characterization of Novel Mycoviruses in Seven Umbelopsis Strains
Source: Viruses. 2022 Oct 25;14(11):2343. doi: 10.3390/v14112343 (PMC9694724; doi:10.3390/v14112343)
Supplement: Supplementary file 1 [file viruses-14-02343-s001.zip › Supplementary Figure S1.pdf]

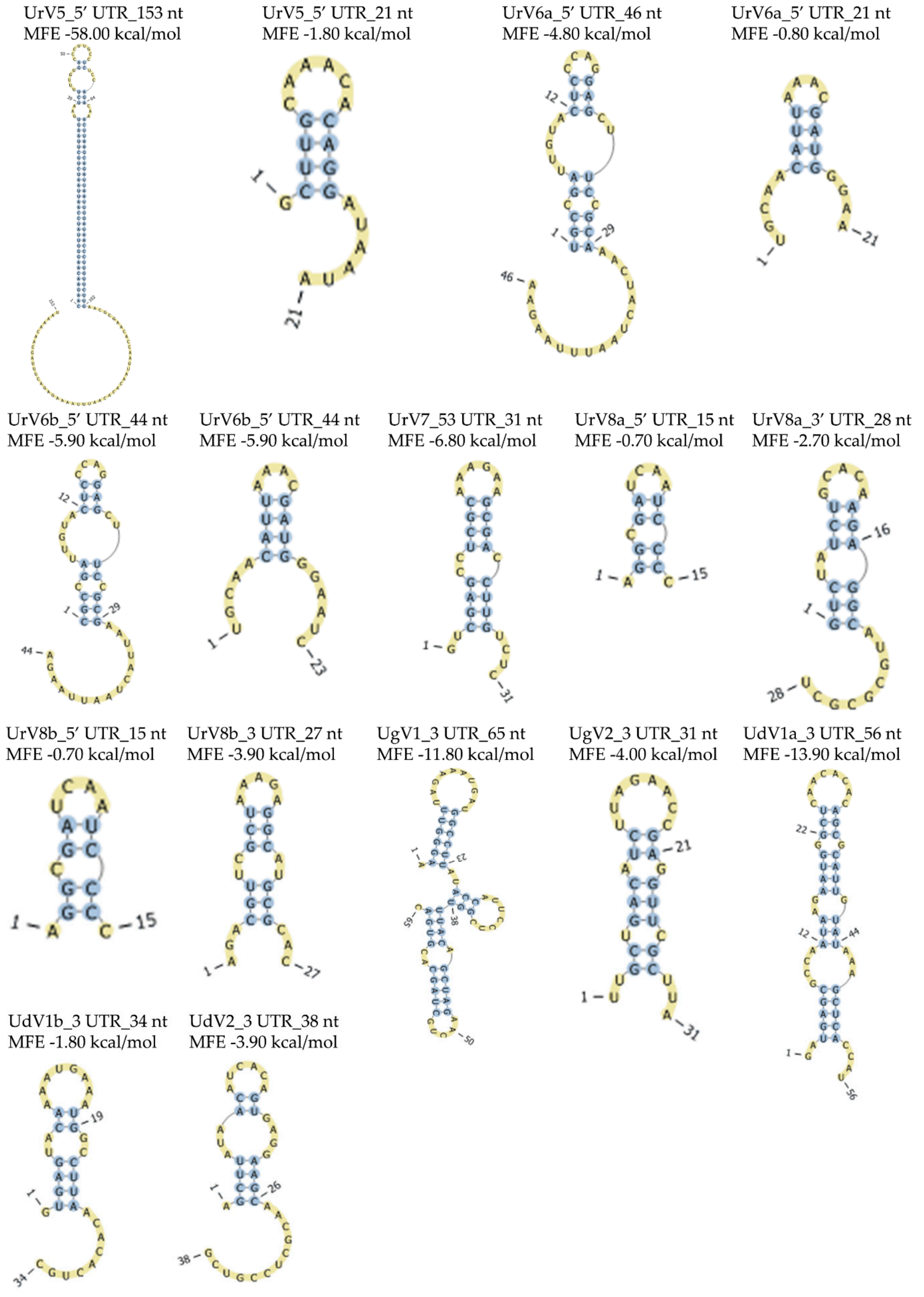

Supplementary Figure S1. The predicted secondary structures of the 5′ and 3′ UTRs of the detected viruses. UrV5, Umbelopsis ramanniana virus 5; UrV6a, Umbelopsis ramanniana virus 6a; UrV6b, Umbelopsis ramanniana virus 6b; UrV7, Umbelopsis ramanniana virus 7; Ur8a, Umbelopsis ramanniana virus 8a; UrV8b, Umbelopsis ramanniana virus 8b; UgV1, Umbelopsis gibberispora virus 1; UgV2, Umbelopsis gibberispora virus 2; UdV1a,

Umbelopsis dimorpha virus 1a; UdV1b, Umbelopsis dimorpha virus 1b; UdV2, Umbelopsis dimorpha virus 2; UdV2; UTR, Untranslated rgion; MFE, minimum free energy.
