# Supplementary material for: Molecular Characterization of Novel Mycoviruses in Seven Umbelopsis Strains
Source: Viruses. 2022 Oct 25;14(11):2343. doi: 10.3390/v14112343 (PMC9694724; doi:10.3390/v14112343)
Supplement: Supplementary file 1 [file viruses-14-02343-s001.zip › Supplementary Figure S2-S7.pdf]

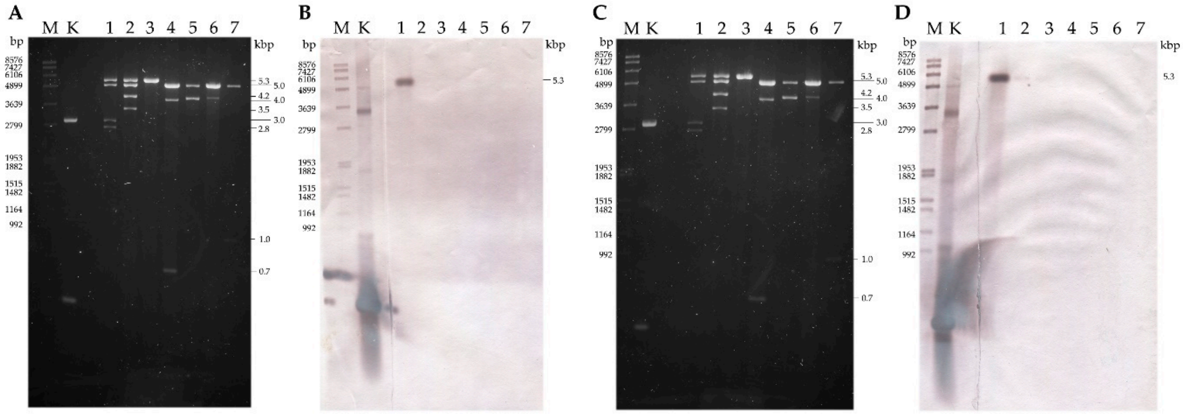

Supplementary Figure S2 Northern blot analysis of dsRNA molecules purified from the mycovirus-harboring *Umbelopsis* strains using the UrV5 CP and RdRp probes, which were amplified with primers presented in Supplementary Table 2. Panels **(A)**, **(C)**, agarose gel electrophoresis of dsRNA molecules purified from the mycovirus-harboring *Umbelopsis* strains. Lane M, DIG-labeled DNA Molecular Weight Marker VII (Roche); Lane K, control plasmid containing the corresponding PCR amplicon of virus genomes; Lane 1, *Umbelopsis ramanniana* NRRL 1296; Lane 2, *Umbelopsis ramanniana* CBS 478.63; Lane 3, *Umbelopsis ramanniana* CBS 243.58; Lane 4, *Umbelopsis gibberispora* CBS 109328; Lane 5, *Umbelopsis angularis* CBS 603.68; Lane 6, *Umbelopsis dimorpha* CBS 110039; Lane 7, *Umbelopsis versiformis* CBS 473.74. Right numbers indicate the sizes (kbp) of the detected dsRNA molecules. Panels (**B**) and (**D**): Northern blot analysis of the dsRNA molecules extracted from *U. ramanniana* NRRL 1296 strain using the UrV5 CP and UrV5 RdRp probes, respectively. Both probes, the UrV5 CP (**B**) and the UrV5 RdRp (**D**) gave strong hybridization signal to the largest, 5.3-kbp band purified from *U. ramanniana* NRRL 1296 strain, as well as the *Bgl*II digested control plasmid, which contains the corresponding PCR amplicon of the UrV5 CP and UrV5 RdRp probe sequences.

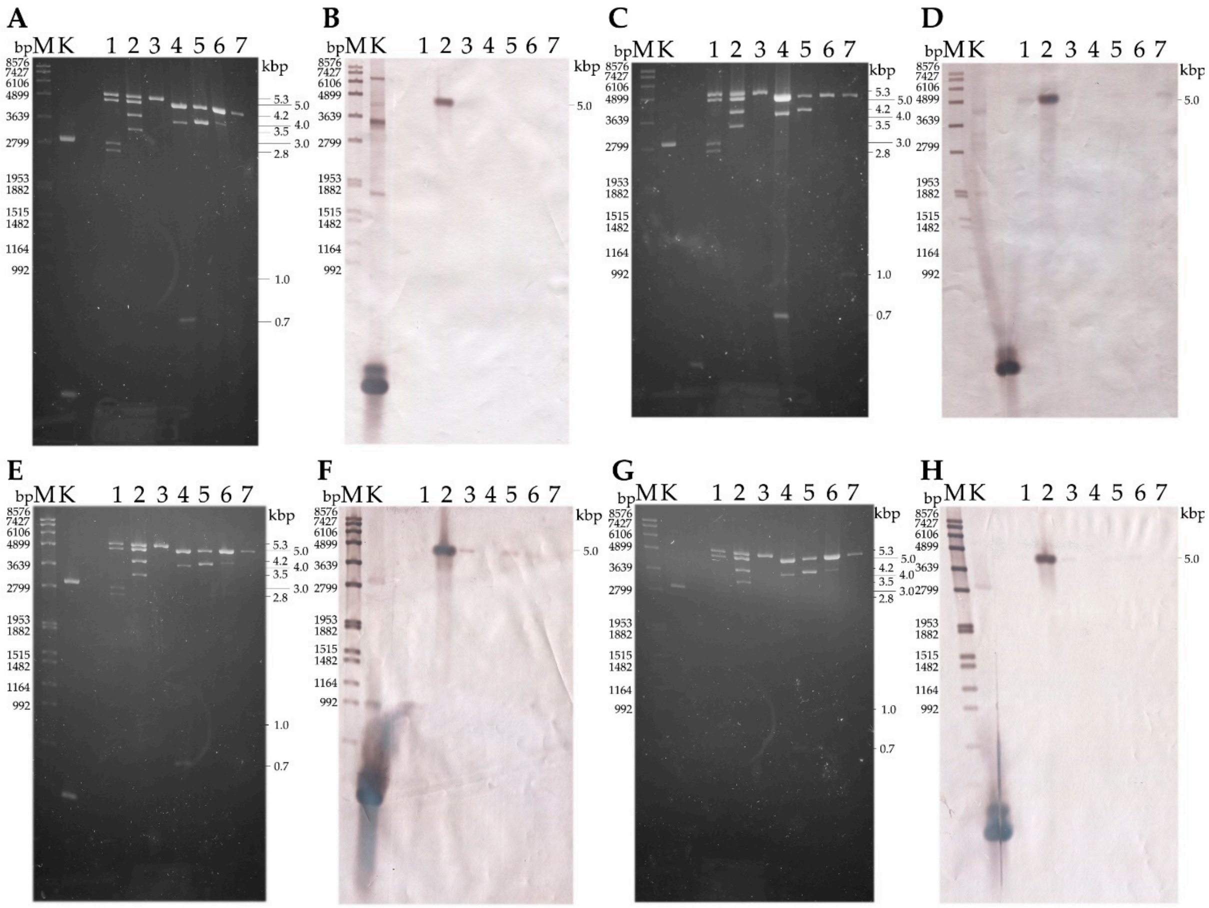

Supplementary Figure S3 Northern blot analysis of dsRNA molecules purified from the mycovirus-harboring *Umbelopsis* strains using the UrV7 and UrV8a CP and RdRP probes, which were amplified with primers presented in Supplementary Table 2. Panels **(A)**, **(C)**, **(E)** and **(G)**: agarose gel electrophoresis of dsRNA molecules purified from the mycovirus-harboring *Umbelopsis* strains. Lane M, DIG-labeled DNA Molecular Weight Marker VII (Roche); Lane K, control plasmid containing the corresponding PCR amplicon of virus genomes; Lane 1, *Umbelopsis ramanniana* NRRL 1296; Lane 2, *Umbelopsis ramanniana* CBS 478.63; Lane 3, *Umbelopsis ramanniana* CBS 243.58; Lane 4, *Umbelopsis gibberispora* CBS 109328; Lane 5, *Umbelopsis angularis* CBS 603.68; Lane 6, *Umbelopsis dimorpha* CBS 110039; Lane 7, *Umbelopsis versiformis* CBS 473.74. Right numbers indicate the sizes (kbp) of the detected dsRNA molecules. Panels (**B**), (**D**), **(F)** and **(H)**: Northern blot analysis of the dsRNA molecules extracted from *U. ramanniana* CBS 478.63 strain using the UrV7 CP, UrV7 RdRp, UrV8 CP and UrV8 RdRp probes, respectively. Panels **(B)** and **(D)**, the UrV7 CP probes gave strong hybridization signal to the 5.0-kbp band purified from *U. ramanniana* CBS 478.63 strain, while the UrV7 RdRp probes gave strong hybridization signal to the 5.0-kbp band purified from *U. ramanniana* CBS 478.63 strain but also hybridized to the 5.0-kbp band extracted from *U. ramanniana* NRRL 1296 and CBS 473.74 strains. Panels **(F)** and **(H)**, the UrV8a CP probes gave strong hybridization signal to the 5.0-kbp band purified from *U. ramanniana* CBS 478.63 strain, but also gave weak hybridization signal to the 5.0-kbp band purified from *U. ramanniana* CBS 243.58, *U. angularis* CBS 603.68 and *U. dimorpha* CBS 110039 strains. The UrV8a RdRp probes gave strong hybridization signal to the 5.0-kbp band purified from *U. ramanniana* CBS 478.63 strain, but also gave hybridization signal to the 5.0-kbp band purified from *U. ramanniana* CBS 243.58 strain.

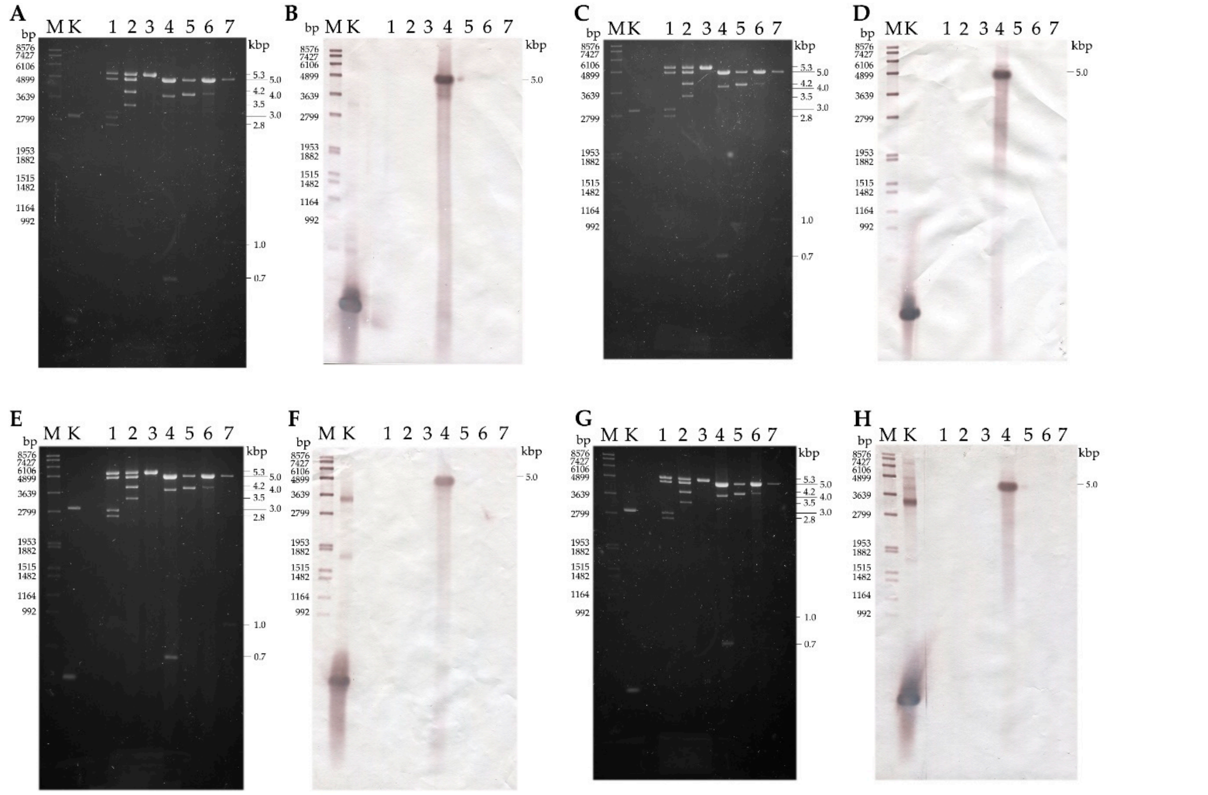

Supplementary Figure S4 Northern blot analysis of dsRNA molecules purified from the mycovirus-harboring *Umbelopsis* strains using the UgV1 and UgV2 CP and RdRo probes, which were amplified with primers presented in Supplementary Table 2. Panels **(A)**, **(C)**, **(E)** and **(G)** agarose gel electrophoresis of dsRNA molecules purified from the mycovirus-harboring *Umbelopsis* strains. Lane M, DIG-labeled DNA Molecular Weight Marker VII (Roche); Lane K, control plasmid containing the corresponding PCR amplicon of virus genomes; Lane 1, *Umbelopsis ramanniana* NRRL 1296; Lane 2, *Umbelopsis ramanniana* CBS 478.63; Lane 3, *Umbelopsis ramanniana* CBS 243.58; Lane 4, *Umbelopsis gibberispora* CBS 109328; Lane 5, *Umbelopsis angularis* CBS 603.68; Lane 6, *Umbelopsis dimorpha* CBS 110039; Lane 7, *Umbelopsis versiformis* CBS 473.74. Right numbers indicate the sizes (kbp) of the detected dsRNA molecules. Panels (**B**), (**D**), **(F)** and **(H)** Northern blot analysis of the dsRNA molecules extracted from *U. gibberispora* CBS 109328 strain using the UgV1 CP, UgV1 RdRp, UgV2 CP and UgV2 RdRp probes, respectively. Both probes, the UgV1 CP (**B**) and the UgV1 RdRp (**D**) gave strong hybridization signal to the largest, 5.0-kbp band purified from *U. gibberispora* CBS 109328 strain. (**F, H**) The UgV2 CP and RdRp probes gave strong hybridization signal also to the 5.0-kbp band extracted form the *U. gibberispora* CBS 109328 izolates.

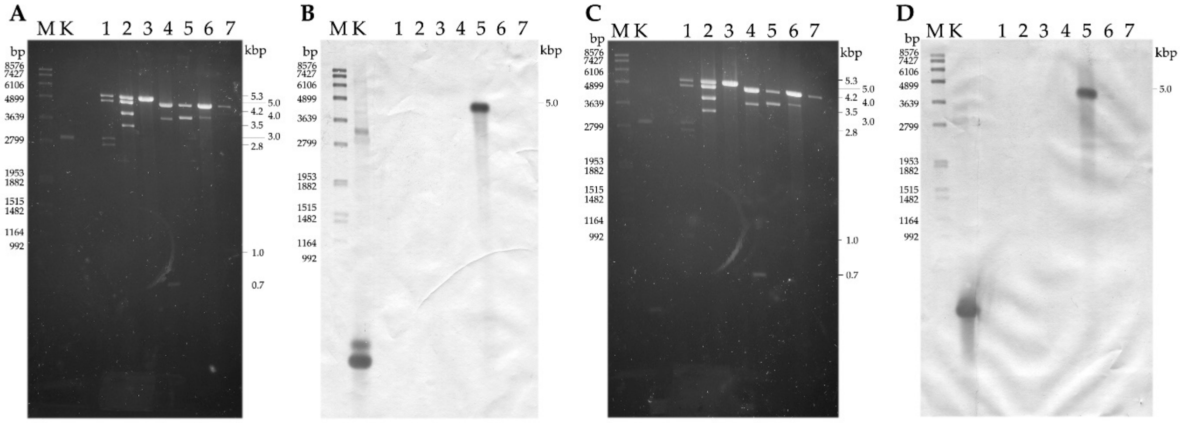

Supplementary Figure S5 Northern blot analysis of dsRNA molecules purified from the mycovirus-harboring *Umbelopsis* strains using the UrV8b CP and RdRp probes, which were amplified with primers presented in Supplementary Table 2. Panels **(A)**, **(C)**, agarose gel electrophoresis of dsRNA molecules purified from the mycovirus-harboring *Umbelopsis* strains. Lane M, DIG-labeled DNA Molecular Weight Marker VII (Roche); Lane K, control plasmid containing the corresponding PCR amplicon of virus genomes; Lane 1, *Umbelopsis ramanniana* NRRL 1296; Lane 2, *Umbelopsis ramanniana* CBS 478.63; Lane 3, *Umbelopsis ramanniana* CBS 243.58; Lane 4, *Umbelopsis gibberispora* CBS 109328; Lane 5, *Umbelopsis angularis* CBS 603.68; Lane 6, *Umbelopsis dimorpha* CBS 110039; Lane 7, *Umbelopsis versiformis* CBS 473.74. Right numbers indicate the sizes (kbp) of the detected dsRNA molecules. Panels (**B**) and (**D**): Northern blot analysis of the dsRNA molecules extracted from *U. angularis* CBS 603.68 using the UrV8b CP and UrV8b RdRp probes, respectively. Both probes, the UrV8b CP (**B**) and the UrV8b RdRp (**D**) gave strong hybridization signal to the largest, 5.0-kbp band purified from *U. angularis* CBS 603.68 strain.

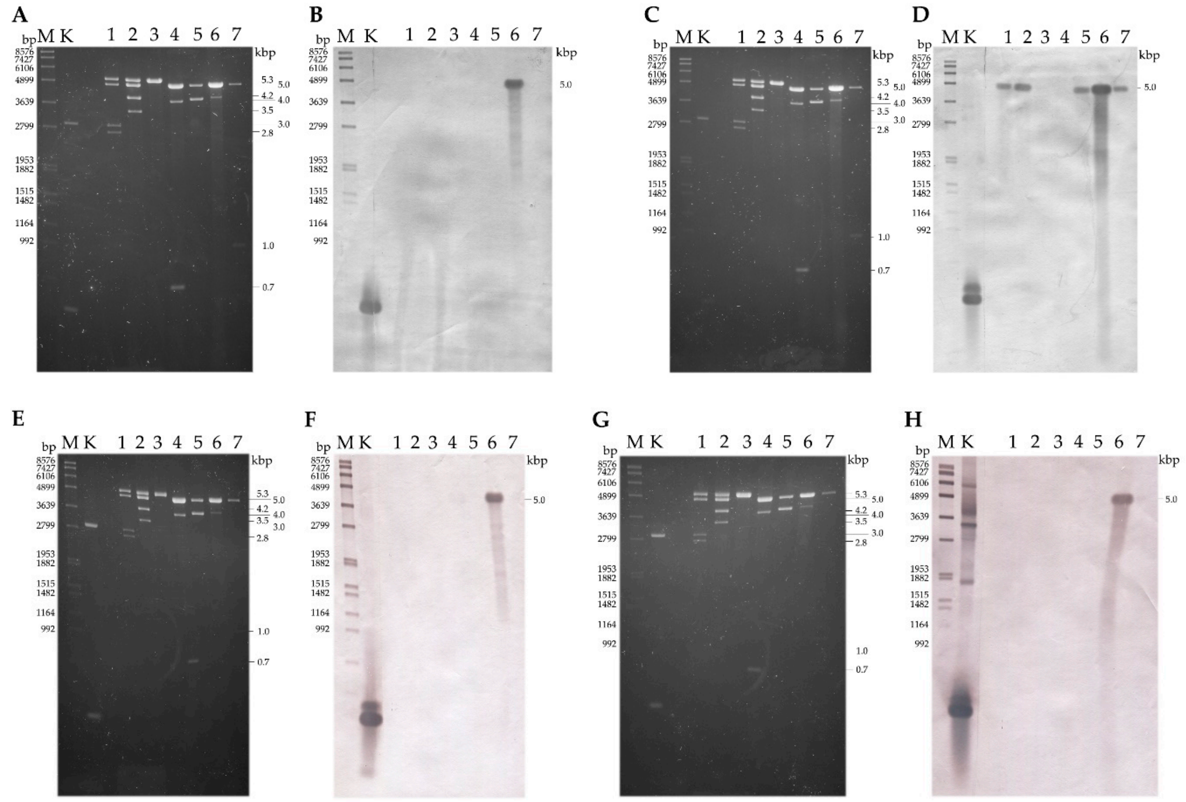

Supplementary Figure S6 Northern blot analysis of dsRNA molecules purified from the mycovirus-harboring *Umbelopsis* strains using the UdV1a and UdV2 CP and RdRp probes, which were amplified with primers presented in Supplementary Table 2. Panels **(A)**, **(C)**, **(E)** and **(G)** agarose gel electrophoresis of dsRNA molecules purified from the mycovirus-harboring *Umbelopsis* strains. Lane M, DIG-labeled DNA Molecular Weight Marker VII (Roche); Lane K, control plasmid containing the corresponding PCR amplicon of virus genomes; Lane 1, *Umbelopsis ramanniana* NRRL 1296; Lane 2, *Umbelopsis ramanniana* CBS 478.63; Lane 3, *Umbelopsis ramanniana* CBS 243.58; Lane 4, *Umbelopsis gibberispora* CBS 109328; Lane 5, *Umbelopsis angularis* CBS 603.68; Lane 6, *Umbelopsis dimorpha* CBS 110039; Lane 7, *Umbelopsis versiformis* CBS 473.74. Right numbers indicate the sizes (kbp) of the detected dsRNA molecules. Panels (**B**), (**D**), **(F)** and **(H)** Northern blot analysis of the dsRNA molecules extracted from *U. ramanniana* NRRL 1296 strain using the UdV1a CP, UdV1a RdRp, UdV2 CP and UdV2 RdRp probes, respectively. UdV1a CP probe **(B)** gave strong hybridization signal to the 5.0-kbp band purified from *U. dimorpha* CBS 110039 strain, while UdV1a RdRp probe **(D)** gave also hybridization signal beside the 5.0-kbp band purified from *U. dimorpha* CBS 110039 to 5.0-kbp band extracted fom *U. ramanniana* NRRL 1296, *U. ramanniana* CBS 478.63, *U. angularis* CBS 603.68 and *U. versiformis* CBS 473.74 strains. (**F, H**) The UdV2 CP and RdRp probes gave strong hybridization signal only to the 5.0-kbp band extracted form the *U. dimorpha* CBS 110039 izolates.

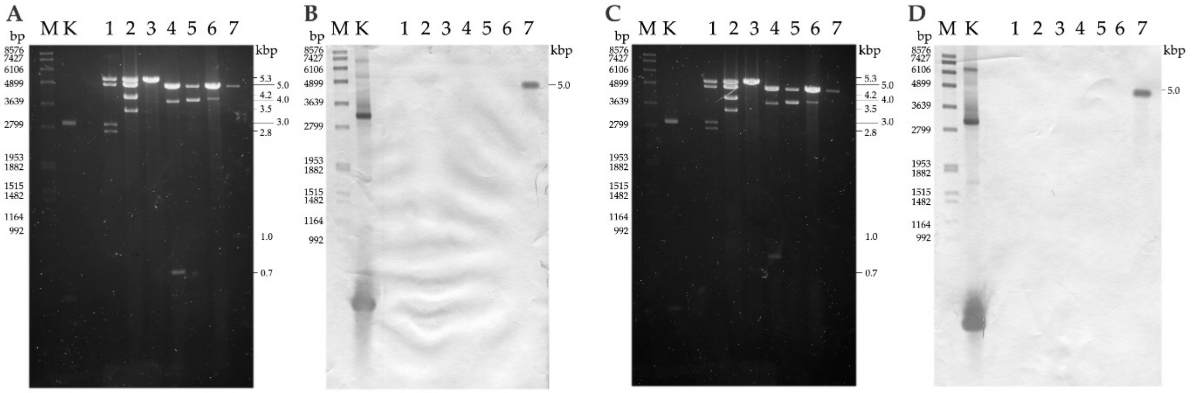

Supplementary Figure S7 Northern blot analysis of dsRNA molecules purified from the mycovirus-harboring Umbelopsis strains using the UdV1b CP and RdRp probes, which were amplified with primers presented in Supplementary Table 2. Panels **(A)**, **(C)**, agarose gel electrophoresis of dsRNA molecules purified from the mycovirus-harboring *Umbelopsis* strains. Lane M, DIG-labeled DNA Molecular Weight Marker VII (Roche); Lane K, control plasmid containing the corresponding PCR amplicon of virus genomes; Lane 1, *Umbelopsis ramanniana* NRRL 1296; Lane 2, *Umbelopsis ramanniana* CBS 478.63; Lane 3, *Umbelopsis ramanniana* CBS 243.58; Lane 4, *Umbelopsis gibberispora* CBS 109328; Lane 5, *Umbelopsis angularis* CBS 603.68; Lane 6, *Umbelopsis dimorpha* CBS 110039; Lane 7, *Umbelopsis versiformis* CBS 473.74. Right numbers indicate the sizes (kbp) of the detected dsRNA molecules. Panels (**B**) and (**D**): Northern blot analysis of the dsRNA molecules extracted from *U. versiformis* CBS 473.74 using the UdV1b CP and UdV1b RdRp probes, respectively. Both probes, the UdV1b CP (**B**) and the UrV8b RdRp (**D**) gave strong hybridization signal to the 5.0-kbp band purified from *U. versiformis* CBS 473.74 strain.
