# Supplementary material for: Molecular Characterization of Novel Mycoviruses in Seven Umbelopsis Strains
Source: Viruses. 2022 Oct 25;14(11):2343. doi: 10.3390/v14112343 (PMC9694724; doi:10.3390/v14112343)
Supplement: Supplementary file 1 [file viruses-14-02343-s001.zip › Supplementary Table S1.pdf]

Supplementary Table S1 *Umbelopsis* strains investigated in this study.

Species name	Collection number	SZMC code	Substrate/Origin	Presence of dsRNAs	References	Accession number of ITS sequence	Accession number of MCM7 sequence
*Mucor lusitanicus**	CBS 277.47	-	-	-	-	-	MF495132.1
Umbelopsis angularis	CBS 603.68	11252	Soil / Baarn, Netherlands	+	[5]	MH859191.1	MF417301.1
Umbelopsis autotrophica	CBS 310.93	11276	Soil / UK	-	[5]	HQ630285	-
*Umbelopsis changbaiensis**	CGMCC 3.16317	-	-	-	-	-	MF417319.1
Umbelopsis dimorpha	CBS 110039	22797	Soil / New Zealand	+	[5]	AJ580917	MF417365.1
*Umbelopsis fusiformis**	CBS 385.85	-	-	-	-	KC489500.1	MF417359.1
Umbelopsis gibberispora	CBS 109328	23390	*Fagus crenata* / Japan	+	[5]	AJ495442	MF417364.1
Umbelopsis isabellina	FSU 909	11324	-	-	[5]	-	-
Umbelopsis isabellina	FSU 910	11323	-	-	[5]	HQ630335	-
Umbelopsis isabellina	FSU 922	11335	-	-	[5]	-	-
Umbelopsis isabellina	FSU 923	11319	-	-	[5]	-	-
Umbelopsis isabellina	FSU 924	11325	-	-	[5]	-	-
Umbelopsis isabellina	FSU 925	11290	-	-	[5]	-	-
Umbelopsis isabellina	FSU 926	11291	-	-	[5]	-	-
Umbelopsis isabellina	FSU 954	11322	-	-	[5]	-	-
Umbelopsis isabellina	FSU 1716	11292	-	-	[5]	-	-
Umbelopsis isabellina	FSU 2831	11293	-	-	[5]	-	-
Umbelopsis isabellina	FSU 2892	11321	-	-	[5]	-	-

Umbelopsis isabellina	NRRL 1757	11076	Soil / Wisconsin, USA	-	[5]	HQ630284	-
Umbelopsis longicollis	CBS 209.32	11208	Sandy loam / Victoria, Australia	-	[5]	HQ630287	MF417368.1
Umbelopsis ovata	CBS 499.82	22674	*Isopogon ceratophyllus* / Victoria, Australia	-	[5]	AJ495429	MF417361.1
Umbelopsis ramanniana	NRRL 1296	11078	- / Wisconsin, USA	+	[5]	HQ630289	-
Umbelopsis ramanniana	CBS 243.58	27285	Man, skin between toes / Netherlands	+	In this study	-	-
Umbelopsis ramanniana	CBS 478.63	27286	*Amanita sp.* / Netherlands	+	In this study	-	-
Umbelopsis ramanniana	CBS 101226	27287	Soil, wood scraps / California	-	In this study	JN206383.1	-
Umbelopsis ramanniana	CBS 110437	27288	Soil / Netherlands	-	In this study	-	-
Umbelopsis ramanniana	CBS 113564	27289	Dead isopod under bark of *Salix* / Netherlands	-	In this study	-	-
Umbelopsis ramanniana	CBS 219.47	27290	*Lactarium deliciosus* / Netherlands	-	In this study	MF417293.1	-
Umbelopsis swartii	CBS 868.85	23388	Soil under *Eucalyptus regnans* / Victoria, Australia	-	[5]	AJ495444, MH861920.1	MF417363.1
Umbelopsis versiformis	CBS 150.81	23387	Root (*Quercus borealis*) / Virginia	-	[5]	AJ495434, MH861311.1	-
Umbelopsis versiformis	CBS 473.74	21866	Poor quality soil / Victoria, Australia	+	[5]	AJ495433	MF417360.1
Umbelopsis vinacea	CBS 222.29	11234	- / USSR	-	[5]	HQ630286.1	-
Umbelopsis vinacea[*]	CBS 212.32	-	-	-	-	MH855292.1	MF417356.1

Umbelopsis vinacea	FSU 2701	11316	-	-	[5]	HQ630335	-
*Umbelopsis westeae**	CBS 870.85	-	-	-	-	MH861922.1	MF417366.1

SZMC: Szeged Microbiology Collection, Hungary; CBS: Centraalbureau voor Schimmelcultures, Baarn, The Netherlands; NRRL: Agricultural Research Service Culture Collection, USA; FSU: *Friedrich Schiller University, Jena*, Germany.

*These strains were used only for the construction of the phylogeny of the *Umbelopsis* species inferred from the ITS and MCM7 sequences.
