# Supplementary material for: Molecular Characterization of Novel Mycoviruses in Seven Umbelopsis Strains
Source: Viruses. 2022 Oct 25;14(11):2343. doi: 10.3390/v14112343 (PMC9694724; doi:10.3390/v14112343)
Supplement: Supplementary file 1 [file viruses-14-02343-s001.zip › Supplementary Table S2.pdf]

Table S2. melléklet: List of primers applying for amplification of cDNAs and hybridization probes.

Primer*	Sequence (5′ - 3′)	Application	Reference
PC3-T7 loop	p- GGATCCCGGGAATTCGGTAATACG ACTCACTATATTTTTATAGTGAGTCGTATTA-OH	self-priming anchor primer for cDNA synthesis	[8]
PC2	p-CCGAATTCCCGGGATCC	Amplification of cDNA	[8]
*U. angularis*_2F	CGTTATATTGCTCATCGTCTTAGG	Specific amplification of 4.0-kbp fragment from *U. angularis*	In this study
*U. angularis*_2R	ACTGAAACTAGAAATCGGTAGG		
UrV5_CP_F	CTGGCTGGAGTGAATAACTTGG	Amplification of probe for UrV5 CP	In this study
UrV5_CP_R	GTCATCAAATTGAGATGCACAG		
UrV5_RdRp_F	CCGTGGGTCTACAAAGCCTG	Amplification of probe for UrV5 RdRp	In this study
UrV5_RdRp_R	CGTATTCCTATGCCCTGACC		
UrV6a_CP_F	ACTGCTTACTCCGGGTTCCA	Amplification of probe for UrV6a CP	In this study
UrV6a_CP_R	GCCCTAACTTGTTCAAAGTAACTG		
UrV6a_RdRp_F	CAAATGCGTCCCATCAAGCC	Amplification of probe for UrV6a RdRp	In this study
UrV6a_RdRp_R	GCCAATGCCTTCTCCTTTGC		
UrV6b_CP_F	ATTCTGTGCGTATGTTCTGG	Amplification of probe for UrV6b CP	In this study
UrV6b_CP_R	GAGAGTATGCGGAAGTGCCT		
UrV6b_RdRp_F	AACGAAGGAGGGAGGTATGG	Amplification of probe for UrV6b RdRp	In this study
UrV6b_RdRp_R	GGTTATGAAAGGCTCCAAGTAGG		
UrV7_CP_F	TACATCCTCTAGACTCCACAATGC	Amplification of probe for UrV7 CP	In this study

Primer*	Sequence (5′ - 3′)	Application	Reference
UrV7_CP_R	CATTGGAGCATCAGTAATCAC		
UrV7_RdRp_F	TCACCCAGACACAGTAAATATAGC	Amplification of probe for UrV7 RdRp	In this study
UrV7_RdRp_R	TACCGACAGGGAACTCATTAGG		
UrV8a_CP_F	ATTTACCTGCCGACTACTCTG	Amplification of probe for UrV8a CP	In this study
UrV8a_CP_R	AAACGGTCCTCCATTCAGTTCG		
UrV8a_RdRp_F	TCAAAGATTTACCTAAGCAACGGG	Amplification of probe for UrV8a RdRp	In this study
UrV8a_RdRp_R	GGCCTAGCCTTATTGCCTACAG		
UrV8b_CP_F	TGACACTAGTAGAATGGAAAGCAC	Amplification of probe for UrV8b CP	In this study
UrV8b_CP_R	TGCAGCCCAAGTTTGGTACTC		
UrV8b_RdRp_F	TAGACGGTGAAGAAAGGATTAAGG	Amplification of probe for UrV8b RdRp	In this study
UrV8b_RdRp_R	AATTTCCTTGGGTTCTCACGA		
UgV1_CP_F	TTCCAGCCTTTGCCATCTTACC	Amplification of probe for UgV1 CP	In this study
UgV1_CP_R	GCGTTGACACTCCGTACTACTG		
UgV1_RdRp_F	TGGGCTAATGCTGTGGTTCG	Amplification of probe for UgV1 RdRp	In this study
UgV1_RdRp_R	GCTGCCTTGAACAATTTGATCG		
UgV2_CP_F	ACCACCGCTACGAGATTGAG	Amplification of probe for UgV2 CP	In this study
UgV2_CP_R	AGAGAACCTGGCTAATACTATGCT		
UgV2_RdRp_F	GACGCATACCTTGAAGCATACC	Amplification of probe for UgV2 RdRp	In this study
UgV2_RdRp_R	AGCCTGATATTCATCTTCTTAGCG		
UdV1a_CP_F	TCGCTCTTGATGATATTGATTGGC	Amplification of probe for UdV1a CP	In this study
UdV1a_CP_R	TTTCCGGTTGCGACACTTATACAC		

Primer*	Sequence (5′ - 3′)	Application	Reference
UdV1a_RdRp_F	TGGGTGGTGTGATGTTATGG	Amplification of probe for UdV1a RdRp	In this study
UdV1a_RdRp_R	ATATCTTGATGGTACTGGCTATGC		
UdV2_CP_F	ATGTATAGTGATGATTCGGGAACC	Amplification of probe for UdV2 CP	In this study
UdV2_CP_R	TGACGTAATCAACAATCCAAGACC		
UdV2_RdRp_F	TGCGAAGATGTATTACCAAACGAG	Amplification of probe for UdV2 RdRp	In this study
UdV2_RdRp_R	GTGACCCTTGTAGCAGTAGTTGTG		
UdV1b_CP_F	TTTCACCAACAAGAGCCAGAG	Amplification of probe for UdV1b CP	In this study
UdV1b_CP_R	ATCGTAAGCGTCTGTGAAAGTACC		
UdV1b_RdRp_F	TTTACGGCACAGGAAGTATGG	Amplification of probe for UdV1b RdRp	In this study
UdV1b_RdRp_R	AGTCAACCTGCCCAACATCC		

*F and R indicating forward and reverse primers, respectively.
