# Supplementary material for: Molecular Characterization of Novel Mycoviruses in Seven Umbelopsis Strains
Source: Viruses. 2022 Oct 25;14(11):2343. doi: 10.3390/v14112343 (PMC9694724; doi:10.3390/v14112343)
Supplement: Supplementary file 1 [file viruses-14-02343-s001.zip › Supplementary Table S3.pdf]

Supplementary Table S3. Amino acid sequence identities of the UrV5 CP, UrV5 RdRp, UrV6a CP, UrV6a RdRp, UrV7 CP, UrV7 RdRp, UrV8a CP, UrV8a RdRp, UrV6b CP, UrV6b RdRp, UgV1 CP, UgV1 RdRp, UgV2 CP, UgV2 RdRp, UgV8b CP, UgV8b RdRp, UdV1a CP, UdV1b RdRp, UdV2 CP, UdV2 RdRp and UdV1b CP, UdV1b RdRp with the most similar sequences deduced by the BLAST search of the NCBI database.

Virus	Virus name	Accession number	Identity (%)	E-Value	Query Coverage (%)
**UrV5 CP**	Totiviridae sp. H4Bulk46262_000002 HP	QDH90455.1	29,47	5e-107	98
	Totiviridae sp. H1Rhizo26FD511_000002 HP	QDH86838.1	33,28	8e-84	67
	Totiviridae sp. H4Bulk46501_000002 HP	QDH87366.1	31,56	5e-65	54
	Totiviridae sp. H1BulkLitter51292_000002 HP	QDH87753.1	28,73	1e-16	40
	Erysiphales associated totivirus 24 CP	QIP68043.1	23,74	3e-13	62
**UrV5 RdRp**	Totiviridae sp. RdRp	QDH87752.1	52,89	0	99
	Totiviridae sp. RdRp	QDH86837.1	54,85	0	92
	Totiviridae sp. RdRp	QDH87365.1	58,65	0	78
	Totiviridae sp. RdRp	QDH90454.1	57,79	0	74
	Totiviridae sp. RdRp	QDH91324.1	56,20	6e-142	57
**UrV6a CP**	Totiviridae sp. H4Bulk46262_000002 HP	QDH90455.1	31,20	6e-129	99
	Totiviridae sp. H1Rhizo26FD511_000002 HP	QDH86838.1	37,21	2e-100	68
	Totiviridae sp. H4Bulk46501_000002 HP	QDH87366.1	31,03	5e-84	84
	Phytophthora condilina RNA virus 2 CP	QTT60992.1	26,06	3e-11	32
	Totiviridae sp. H1BulkLitter51292_000002 HP	QDH87753.1	27,49	3e-11	41
**UrV6a RdRp**	Totiviridae sp. RdRp	QDH87752.1	60,11	0	96
	Totiviridae sp. RdRp	QDH86837.1	60,69	0	88
	Totiviridae sp. RdRp	QDH87365.1	59,24	0	77
	Totiviridae sp. RdRp	QDH90454.1	63,47	0	73
	Totiviridae sp. RdRp	QDH91324.1	55,33	9e-160	60
**UrV7 CP**	Mucor hiemalis virus 4 CP	CAE9672667.1	46,23	0	98
	Trichoderma koningiopsis totivirus 1 CP	QGA70770.1	45,55	0	99

Virus	Virus name	Accession number	Identity (%)	E-Value	Query Coverage (%)
	Wuhan insect virus 26 HP1	YP_009342427.1	43,36	3e-150	78
	Wuhan insect virus 27 HP 1	YP_009342433.1	38,01	3e-149	95
	Totiviridae sp. H4Bulk46889_000002 HP	QDH88821.1	37,67	4e-149	96
**UrV7 RdRp**	Umbelopsis ramanniana virus 4 RdRp	VUD77425.1	86,26	0	100
	Trichoderma koningiopsis totivirus 1 RdRp	QGA70771.1	60,48	0	91
	Wuhan insect virus 26 HP 2	YP_009342428.1	56,04	0	96
	Conidiobolus heterosporus totivirus 1 RdRp	QUE40494.1	49,17	0	95
	Wuhan insect virus 27 HP 2	YP_009342434.1	48,78	0	95
**UrV8a CP**	Trichoderma koningiopsis totivirus 1 CP	QGA70770.1	48,68	0	99
	Mucor hiemalis virus 4 CP	CAE9672667.1	47,88	0	99
	Wuhan insect virus 26 HP 1	YP_009342427.1	44,36	3e-166	78
	Wuhan insect virus 27 HP 1	YP_009342433.1	38,82	1e-153	98
	Totiviridae sp. H4Bulk46889_000002 HP	QDH88821.1	37,52	3e-153	96
**UrV8a RdRp**	Umbelopsis ramanniana virus 4 RdRp	VUD77425.1	65,26	0	96
	Trichoderma koningiopsis totivirus 1 RdRp	QGA70771.1	62,18	0	91
	Wuhan insect virus 26 HP 2	YP_009342428.1	55,84	0	99
	Conidiobolus heterosporus totivirus 1 RdRp	QUE40494.1	52,20	0	97
	Wuhan insect virus 27 HP 2	YP_009342434.1	50,90	0	96
**UrV6b CP**	Totiviridae sp. H4Bulk46262_000002 HP	QDH90455.1	31,77	1e-130	99
	Totiviridae sp. H1Rhizo26FD511_000002 HP	QDH86838.1	35,68	7e-100	68
	Totiviridae sp. H4Bulk46501_000002 HP	QDH87366.1	30,95	5e-82	84
	Phytophthora condilina RNA virus 2 CP	QTT60992.1	24,52	4e-12	46

Virus	Virus name	Accession number	Identity (%)	E-Value	Query Coverage (%)
	Totiviridae sp. H1BulkLitter51292_000002 HP	QDH87753.1	24,15	6e-08	41
**UrV6b RdRp**	Totiviridae sp. RdRp	QDH87752.1	58,87	0	98
	Totiviridae sp. RdRp	QDH86837.1	59,33	0	91
	Totiviridae sp. RdRp	QDH90454.1	63,29	0	73
	Totiviridae sp. RdRp	QDH87365.1	58,89	0	77
	Totiviridae sp. RdRp	QDH91324.1	55,93	4e-159	59
**UgV1 CP**	Plasmopara viticola lesion ass. toti 3 CP	QGY72631.1	42,13	0	97
	Plasmopara viticola lesion ass.toti 4 CP	QGY72633.1	39,53	2e-165	100
	Xanthophyllomyces dendrorhous virus L1A CP	YP_007697650.1	37,24	2e-155	100
	Rhodosporidiobolus odoratus RNA virus 1 CP	QPH36699.1	39,78	1e-154	95
	Red clover powdery mildew-ass. totivirus 4 CP	BAT62483.1	37,23	2e-148	93
**UgV1 RdRp**	Plasmopara viticola lesion ass. toti 3 RdRp	QGY72630.1	54,39	0	99
	Xanthophyllomyces dendrorhous virus L1A RdRp	YP_007697651.1	53,07	0	98
	Xanthophyllomyces dendrorhous virus L2 RdRp	AFH09416.2	53,07	0	98
	Xanthophyllomyces dendrorhous virus L1B RdRp	YP_009507835.1	51,13	0	98
	Erysiphe necator ass. totivirus 5 RdRp	QJW70333.1	50,40	0	98
**UgV2 CP**	Trichoderma koningiopsis totivirus 1 CP	QGA70770.1	48,46	0	98
	Mucor hiemalis virus 4 CP	CAE9672667.1	47,44	0	98
	Wuhan insect virus 26 HP 1	YP_009342427.1	50,45	0	80
	Totiviridae sp. H4Bulk46889_000002 HP	QDH88821.1	41,16	1e-177	97
	Conidiobolus heterosporus totivirus 1 CP	QUE40493.1	39,29	1e-160	97
	Wuhan insect virus 26 HP 2	YP_009342428.1	57,11	0	97

Virus	Virus name	Accession number	Identity (%)	E-Value	Query Coverage (%)
**UgV2 RdRp**	Trichoderma koningiopsis totivirus 1 RdRp	QGA70771.1	58,63	0	89
	Umbelopsis ramanniana virus 4 RdRp	VUD77425.1	56,14	0	93
	Conidiobolus heterosporus totivirus 1 RdRp	QUE40494.1	52,33	0	95
	Wuhan insect virus 27 HP 2	YP_009342434.1	52,28	0	94
**UrV8b CP**	Trichoderma koningiopsis totivirus 1 CP	QGA70770.1	48,32	0	99
	Mucor hiemalis virus 4 CP	CAE9672667.1	47,73	0	99
	Wuhan insect virus 26 HP 1	YP_009342427.1	43,99	5e-164	78
	Wuhan insect virus 27 HP 1	YP_009342433.1	39,12	1e-156	98
	Totiviridae sp. H4Bulk46889_000002 HP	QDH88821.1	37,99	1e-152	96
**UrV8b RdRp**	Umbelopsis ramanniana virus 4 RdRp	VUD77425.1	64,37	0	96
	Trichoderma koningiopsis totivirus 1 RdRp	QGA70771.1	61,64	0	91
	Wuhan insect virus 26 HP 2	YP_009342428.1	55,35	0	99
	Conidiobolus heterosporus totivirus 1 RdRp	QUE40494.1	51,57	0	97
	Wuhan insect virus 27 HP 2	YP_009342434.1	50,64	0	96
**UdV1a CP**	Mucor hiemalis virus 4 CP	CAE9672667.1	45,76	0	99
	Trichoderma koningiopsis totivirus 1 CP	QGA70770.1	43,75	0	99
	Wuhan insect virus 26 HP 1	YP_009342427.1	43,45	8e-154	80
	Totiviridae sp. H4Bulk46889_000002 HP	QDH88821.1	38,36	5e-147	92
	Conidiobolus heterosporus totivirus 1 CP	QUE40493.1	37,69	2e-142	99
**UdV1a RdRp**	Umbelopsis ramanniana virus 4 RdRp	VUD77425.1	73,50	0	100
	Wuhan insect virus 26 HP 2	YP_009342428.1	54,04	0	100
	Trichoderma koningiopsis totivirus 1 RdRp	QGA70771.1	57,14	0	95

Virus	Virus name	Accession number	Identity (%)	E-Value	Query Coverage (%)
	Conidiobolus heterosporus totivirus 1 RdRp	QUE40494.1	47,79	0	99
	Wuhan insect virus 27 HP 2	YP_009342434.1	46,81	0	99
**UdV2 CP**	Trichoderma koningiopsis totivirus 1 CP	QGA70770.1	49,56	0	99
	Mucor hiemalis virus 4 CP	CAE9672667.1	47,95	0	99
	Wuhan insect virus 26 HP 1	YP_009342427.1	44,63	1e-163	78
	Totiviridae sp. H4Bulk46889_000002 HP	QDH88821.1	36,84	8e-149	96
	Wuhan insect virus 27 HP 1	YP_009342433.1	36,91	8e-146	95
**UdV2 RdRp**	Umbelopsis ramanniana virus 4 RdRp	VUD77425.1	66,88	0	98
	Wuhan insect virus 26 HP 2	YP_009342428.1	56,96	0	99
	Trichoderma koningiopsis totivirus 1 RdRp	QGA70771.1	61,24	0	93
	Conidiobolus heterosporus totivirus 1 RdRp	QUE40494.1	53,95	0	99
	Wuhan insect virus 27 HP 2	YP_009342434.1	50,38	0	99
**UdV1b CP**	Mucor hiemalis virus 4 CP	CAE9672667.1	46,12	0	98
	Trichoderma koningiopsis totivirus 1 CP	QGA70770.1	44,67	0	99
	Wuhan insect virus 26 HP 1	YP_009342427.1	43,62	2e-159	80
	Totiviridae sp. H4Bulk46889_000002 HP	QDH88821.1	37,44	1e-147	93
	Conidiobolus heterosporus totivirus 1 CP	QUE40493.1	37,93	2e-143	93
**UdV1b RdRp**	RdRp Umbelopsis ramanniana virus 4	VUD77425.1	74,66	0	98
	RdRp Trichoderma koningiopsis totivirus 1	QGA70771.1	60,35	0	89
	Wuhan insect virus 26 HP 2	YP_009342428.1	54,68	0	97
	RdRp Conidiobolus heterosporus totivirus 1	QUE40494.1	49,42	0	94
	Wuhan insect virus 27 HP 2	YP_009342434.1	48,57	0	97
